# Exploring the Role of *E. Coli* Nissle 1917 Postbiotics as Antimicrobial and Antioxidant Agents for Enhancing Buffalo Sperm Quality

**DOI:** 10.1007/s12602-026-10932-z

**Published:** 2026-02-16

**Authors:** Mohamed S. Darwish, Wael A. Khalil, Mahmoud A. E. Hassan, Mahmoud Moussa, Noha A. Abou-Zeid, Sameh A. Abdelnour, Asmaa A. El-Awady

**Affiliations:** 1 Dairy Microbiology Laboratory, Dairy Department, Faculty of Agriculture, Mansoura University, Mansoura, 35516 Egypt; 2 Department of Animal Production, Faculty of Agriculture, Mansoura University, Mansoura, 35516 Egypt; 3 Animal Production Research Institute, Agriculture Research Centre, Ministry of Agriculture, Dokki, Giza, 12619 Egypt; 4 Department of Theriogenology, Faculty of Veterinary Medicine, Suez Canal University, Ismailia, 41522 Egypt; 5 Veterinary Medicine Directorate, Mansoura, 35516 Egypt; 6 Department of Animal Production, Faculty of Agriculture, Zagazig University, Zagazig, 44511 Egypt

**Keywords:** *E. Coli* Nissle 1917 postbiotic, Antibacterial, Antioxidant, Antiapoptotic, Sperm buffalo

## Abstract

This study focused on the isolation and comprehensive evaluation of a postbiotic synthesized by *E. coli* Nissle 1917. The research specifically investigated its diverse biological activities, including its potential antibacterial, bacteriostatic, bactericidal effects, and its antioxidant capacity. The primary objective was to determine the postbiotic’s efficacy in reducing semen bacterial load and mitigating oxidative stress, enhancing the quality and structural integrity. The results indicated that *E. coli* Nissle 1917 postbiotic exhibited significant antibacterial activity against *K. pneumoniae*, *S. epidermidis*, and *S. aureus*. The bacteriostatic effect was observed against *K. pneumoniae* (900 µg/mL), *E. coli* (1800 µg/mL), *P. aeruginosa* (1500 µg/mL), *S. aureus* (1200 µg/mL), *S. epidermidis* (900 µg/mL), and *B. subtilis* (1500 µg/mL). The bactericidal effect was most pronounced against *K. pneumoniae* and *S. epidermidis*, which were the most sensitive, with a minimum bactericidal concentration (MBC) of 1000 µg/mL, followed by *S. aureus* (1400 µg/mL), *P. aeruginosa* and *B. subtilis* (1650 µg/mL). The main components of the postbiotic are docosanoic acid, 1,2,3-propanetriyl ester (0.90%), stearic acid, 3-(octadecyloxy)propyl ester (1.24%), 1-methyl-2-pyrrolidineethanol (1.49%), 4(3 H)-pyrimidinone / 1 H-imidazole-4,5-dihydro-2-methyl (0.96% / 0.87%), 10-octadecenoic acid methyl ester and similar C18 fatty acid methyl esters (3.74%), cyclohexanol, 1R-4-acetamido-2,3-cis-epoxy (3.34%), 2-myristynoyl-glycinamide (1.85%), 1-monolinoleoylglycerol trimethylsilyl ether (1.81%), and glycine, N-3,5,7,12-tetrakis(trimethylsiloxy)cholan-24-yl derivative (0.82%). Supplementation with 1000–2000 µg/mL postbiotic significantly improved sperm motility (25.7%, and 29.5%), viability (26.8%, and 34%), membrane integrity (22.2%, and 27.1%), and kinematic parameters compared to the control group (*p* < 0.01, respectively). Postbiotic addition also increased the percentage of live sperm with intact acrosome and reduced the percentages of live and dead sperm with detached acrosome. Postbiotic addition (250, 500, 1000 and 2000 µg/mL) significantly improved total antioxidant capacity (35.6, 36.9, 52 and 63%), reduced oxidative stress markers such as malondialdehyde (6.1, 8.7, 19.5 and 22.9%), and nitric oxide (20, 28.2, 36.4 and 38.9%, respectively) and decreased the percentage of apoptotic sperm (26, 46.5, 48.9 and 51.2%) in post-thawed semen compared to control group (*p* < 0.01). Postbiotic supplementation preserved sperm ultrastructure and enhanced pregnancy rates as well as reduced the bacterial load in post-thawed semen (*p* < 0.05). In summary, the *E. coli* Nissle 1917 postbiotic acts as a crucial protective agent. By exerting antimicrobial, anti-apoptotic, and antioxidant activities, it effectively regulates the semen’s antioxidant status and maintains the quality of cryopreserved buffalo sperm.

## Introduction

The use of probiotics has garnered significant attention over the last two decades due to their ecofriendly, safe, and effective multi-target applications in various fields [1]. Moreover, the rise of antimicrobial resistance has contributed to the increased popularity of probiotics as an alternative to the widespread use of antibiotic drugs [2]. However, despite these benefits, concerns have emerged about the potential for probiotics to serve as reservoirs of antibiotic resistance genes. Studies have found that probiotic *Lactobacillus* strains may carry genes conferring resistance to multiple antibiotics [3], including tetracycline and macrolides. These genes can be horizontally transferred to pathogenic bacteria through conjugation, transformation, and transduction mechanisms [4, 5]. This raises safety considerations, especially in vulnerable populations such as neonates and immunocompromised individuals [5]. Careful screening for antibiotic resistance genes in probiotic strains is crucial to mitigate these risks.

Probiotics are live beneficial microorganisms that can replace or compete with pathogenic bacteria, thereby supporting the colonization of a beneficial bacterial community [6, 7]. These bacteria produce substances that enhance gut health and help in the treatment of many diseases, which in turn supports overall health [1, 8]. The positive impacts may be attributed to the presence of phenolic compounds and other substances that can reduce oxidative stress, inflammation, and apoptosis [9]. Additionally, these substances can enhance immune function and the antioxidative defense system within the body’s cells [9].

Postbiotics, which are the main components produced by probiotics, can be defined as “non-viable bacterial products or metabolic products from microorganisms that have biological activity in the host“ [1]. The numerous postbiotic biomolecules comprise metabolic byproducts of live probiotic bacteria such as secreted proteins/peptides, cell-free supernatants, vitamins, short-chain fatty acids, neurotransmitters, organic acids, bacteriocins, and secreted biosurfactants [10, 11]. Moreover, it contains other components such as flavonoids-derived postbiotics (equol, daidzein, desaminotyrosine, norathyriol), terpenoids-derived postbiotics (paeonolactone glycosides, paeoniflorin, genipin, paeonimetabolin I, II, III), phenolic-derived postbiotics (equol, urolithins, valerolactones, enterolactone, enterodiol, 8-prenylnaringenin), etc [12, 13]. Interestingly, these postbiotics can exert certain functional purposes supported by the integral bacterium, comprising anti-apoptotic, anti-inflammatory, antioxidant, immunomodulatory effects and microbiota modulations [6, 14, 15]. *Escherichia coli* Nissle 1917 (EcN) is a well-established safe probiotic strain that is distinct from pathogenic *E. coli*. Genomic studies have confirmed that EcN lacks genes encoding harmful toxins or virulence factors commonly found in pathogenic strains [16, 17]. Clinically, EcN has been used safely for decades in products like Mutaflor^®^ with no evidence of genotoxicity or infection risks, even in vulnerable populations [18]. The mechanistic basis for using EcN extends beyond its live probiotic form to include the biological activities of its cell-free supernatant (CFS) and extracellular vehicles (EVs). These secreted fractions contain bioactive molecules like organic acids, peptides, and signaling metabolites that modulate host-microbe interactions [1, 3, 18]. They enhance intestinal barrier integrity, suppress inflammatory signaling, and regulate immune responses [6, 19]. EcN helps restore epithelial homeostasis, maintain microbial balance, and offer potential health benefits [7, 16]. Thus, EcN has become widely used for both prevention and treatment of several diseases as a postbiotics bacterium [6, 7, 20]. However, its role in mitigating oxidative stress-induced cryo-injury in the sperm cryopreservation process remains unexplored. Numerous reports have recommended that postbiotics derived from EcN display immunomodulatory actions on the intestinal barrier, leading to the decrease of inflammatory features expression and the augmentation of anti-inflammatory features [6, 7, 20].

Recent findings emphasize that postbiotics, defined as bioactive compounds produced by microbial fermentation, exert significant benefits in livestock reproduction by modulating gut microbiota and systemic immunity [21, 22]. These metabolites improve gut barrier function, reduce inflammation, and enhance antioxidant defenses, which collectively support reproductive health and fertility. For instance, postbiotics from *Saccharomyces cerevisiae* and *Lactobacillus* species have demonstrated improved egg production and reduced pathogen colonization in poultry, as well as enhanced nutrient digestibility and immune modulation in ruminants [23]. These findings reinforce postbiotics as promising alternatives to antibiotics, with sustainable benefits for livestock reproductive performance [24, 25].

Current advances indicate that low concentrations (5%) of *Lactobacillus rhamnosus* PB01 postbiotic do not impair sperm quality, but higher concentrations reduce motility and increase DNA fragmentation, highlighting the need for dose optimization and further research [8]. Supplementation with lactic acid bacteria-based postbiotics improved semen quality and liver health in rabbits, suggesting potential benefits [22]. However, current challenges in postbiotic research include variability in composition and a lack of standardized quantification methods, as no single ‘gold standard’ exists for postbiotic characterization. A study by [26]emphasize the need for tailored analytical techniques and robust clinical trials to ensure consistent production, safety, and efficacy. These limitations underscore the importance of further rigorous studies to refine practical recommendations for reproductive health applications.

Gut microbiota-derived metabolites play a crucial role in maintaining sperm oxidative balance by modulating systemic antioxidant defenses and reducing oxidative stress [27]. These metabolites, such as short-chain fatty acids and antioxidants like glutathione, enhance the host’s ability to neutralize ROS, which can damage sperm through lipid peroxidation, DNA fragmentation, and impaired motility [28]. By influencing key antioxidant enzymes and reducing inflammatory signaling, gut microbiota helps preserve sperm function and vitality, highlighting the integral connection between gut microbial health and male reproductive oxidative homeostasis [29].

Cryopreservation is a crucial technique in assisted reproductive technology that provides a robust strategy for preserving genetic material, conserving endangered species, and enhancing genetic improvement in modern animal breeding [30]. However, the process of freeze-thaw cycles can lead to changes in temperature and exposure of sperm cells to low temperatures [31], resulting in increased generation of oxidative stress and reduced antioxidant defense systems in sperm cells [32]. During cryopreservation, sperm produces an excess of reactive oxygen species (ROS) that leads to lipid peroxidation, membrane damage, apoptosis, and decreased mitochondrial function [31]. This oxidative stress overwhelms the natural antioxidant defenses of sperm, disrupting the balance between oxidation and antioxidation [33]. The cryopreservation process can cause significant damage to sperm, resulting in reduced motility, viability, membrane damage, and an increase in apoptotic sperm. Exposure to low temperatures could promote the growth of certain pathogenic microbes in frozen semen [34]. Specifically, these stresses can lead to increased damage to mitochondria and nuclei, resulting in DNA damage and protein oxidation within the cellular system [35], ultimately reducing fertilizing capacity. To enhance the efficacy of sperm cryopreservation, several antioxidants have been added to neutralize oxidative stress and promote the antioxidant defense system in sperm cells [36]. Based on its biological activity, the authors hypothesized that the addition of postbiotic *E. coli* Nissle 1917 (EcN) could reduce oxidative stress in cryopreserved semen, exerting antimicrobial, anti-apoptotic, and antioxidant activities. This could potentially enhance cryo-resistance and improve post-thawed sperm quality. This study aimed to prepare postbiotic *E. coli* Nissle 1917 and explore their various functional roles, such as potential antibacterial, bacteriostatic, and bactericidal effects, antioxidant activity, and beneficial effects in reducing semen bacteria in buffaloes. Additionally, the study investigated the impact of postbiotics on several parameters of cryopreserved semen, including sperm quality, apoptosis, acrosome status, kinematic parameters, antioxidant status, sperm ultrastructure and *i*n vivo fertility.

## Materials and Methods

### Bacterial Strains, Culture Conditions and Preparation of *E. Coli* Nissle 1917 Postbiotic

**Fig. 1:**
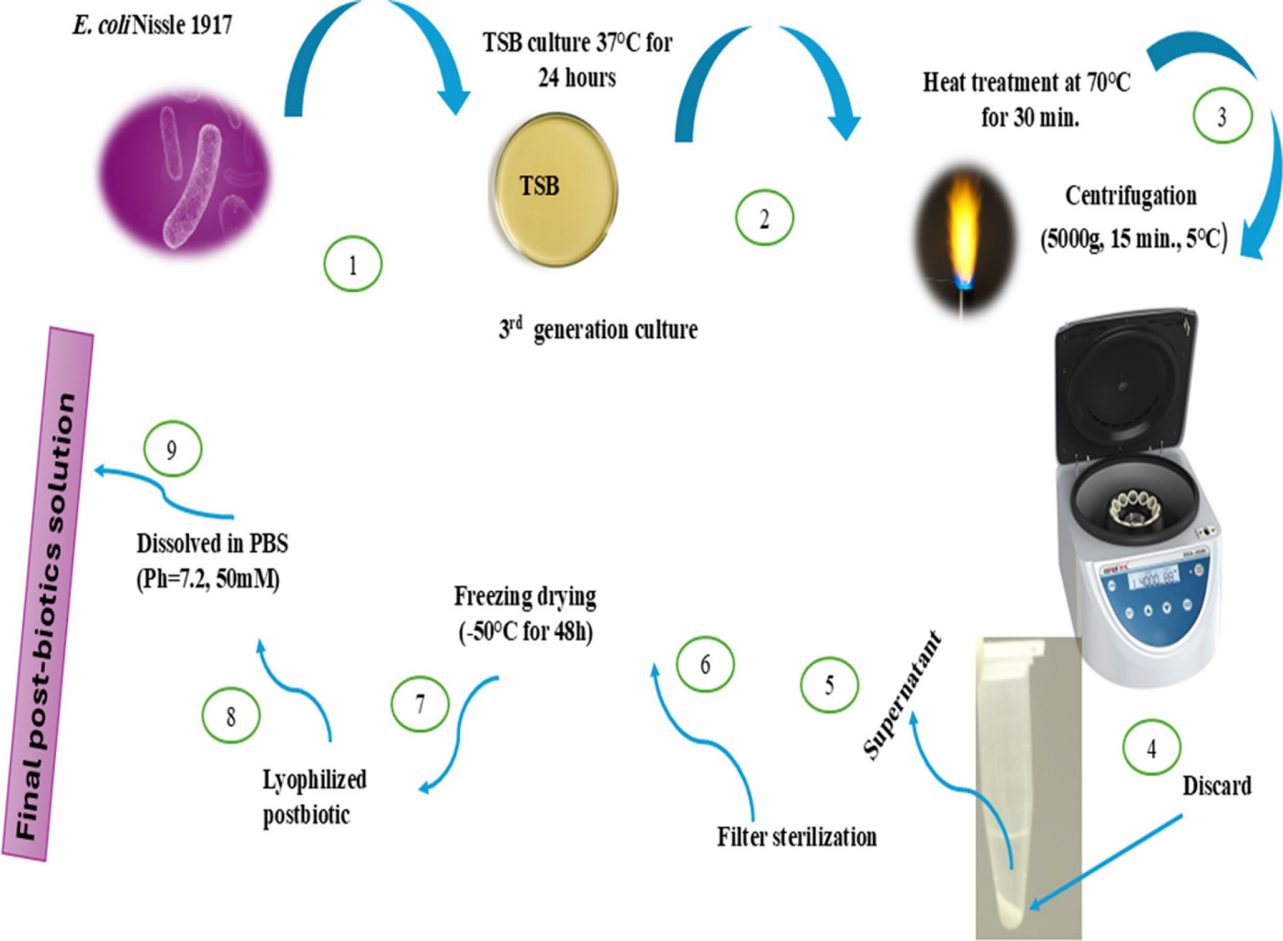
Flowchart of EcN postbiotic preparation

All bacterial strains, including *Escherichia coli* Nissle 1917 (EcN), *Escherichia coli* (BAA 2472), *Pseudomonas aeruginosa* (FML102), *Klebsiella pneumoniae* (FML5), *Staphylococcus aureus* (ATCC 25923), *Staphylococcus epidermidis* (ATCC 12228), and *Bacillus subtilis* (ATCC 6633), were obtained from the Food Microbiology Laboratory, Dairy Department, Faculty of Agriculture, Mansoura University. Each strain was streaked onto nutrient agar plates (BD Difco, NJ, USA) and incubated at 37 ± 1 °C for 24 h. The bacterial cultures were subsequently preserved at − 80 °C for long-term storage. The postbiotic derived from *E. coli* Nissle 1917 (EcN) was prepared using the method described by [37], with slight adjustments. The detailed procedure for postbiotic production is outlined in Fig. 1.

### Antimicrobial Activity of Postbiotic

The antimicrobial activity of different concentrations of postbiotic against *E. coli*, *P. aeruginosa*, *K. pneumoniae*, *S. aureus*, *S. epidermidis*, and *B. subtilis* was assessed using the disc diffusion test as designated by [38]. Briefly, bacterial inocula were prepared from overnight cultures grown on nutrient agar, and colonies were suspended in saline to achieve a turbidity equivalent to 1 × 10^8^ CFU/mL (0.5 McFarland standard). An aliquot part of 100 µL from each suspension was spread evenly onto agar plates. Freshly prepared postbiotic solutions, with doses ranging from 10 to 2000 µg/mL, were then used in the diffusion assay. Chloramphenicol stock solution (10 mg/mL) and sterile water and served as the positive and negative standards, respectively. Postbiotic samples were applied to sterile 8 mm Whatman paper discs, which were carefully placed onto the inoculated Petri dishes and incubated at 37 °C. Inhibition zones were classified as follows: very strong (≥ 30 mm, including disc diameter), strong (≥ 25 mm), moderate (10–20 mm), weak (5–10 mm) and no inhibition if the zone diameter is less than 5 mm. All assays were achieved in triplicate.

### Minimum Inhibitory Concentration (MIC) Determination

Different concentrations of the postbiotic were prepared using Milli-Q water. The MIC of the postbiotic was evaluated against *K. pneumoniae*, *E. coli*, *S. epidermidis*, *S. aureus*, *P. aeruginosa*, and *B. subtilis* using the microtiter plate dilution method, according to [21], with minor modifications. All indicator strains were cultured overnight (24 h) in Nutrient Broth (NB, BD Difco) and subsequently incubated at 37 ± 1 °C for 20–24 h. Microbial cells were collected by centrifugation at 8000 rpm, washed twice with phosphate-buffered saline (PBS), and resuspended in sterile NB to reach a final concentration of 10^8^ CFU/mL. Microtiter plates were then inoculated with 100 µL of each bacterial suspension along with varying concentrations of the postbiotic (300–1800 µg/mL), followed by incubation at 37 °C for 24 h. Optical density was measured at 600 nm using a plate reader (BioTek Teknova, Hollister, CA, USA). The absorbance of blank NB wells was 0.045 ± 0.034, and values exceeding 0.1 were considered positive for bacterial growth. Chloramphenicol at a concentration of 60 µg/mL was employed as the positive control. All trials were shown in triplicate, and outcomes were stated as the standard error of the mean (SEM).

### Subinhibitory Doses of Postbiotic

The bacterial growth curve was generated in the attendance of varying concentrations of the postbiotic using six bacterial strains (*S. epidermidis*, *S. aureus*, *K. pneumoniae*, *P. aeruginosa*, *E. coli*, and *B. subtilis*) to determine the subinhibitory dose. Each strain was cultured overnight in nutrient broth, reaching approximately 10^8^ CFU/mL, and this concentration was used as the inoculum for subsequent assays. An aliquot of 100 µL, together with specific postbiotic concentrations (650–850 µg/mL for *K. pneumoniae*, 1550–1750 µg/mL for *E. coli*, 1250–1450 µg/mL for *P. aeruginosa*, 950–1150 µg/mL for *S. aureus*, 650–850 µg/mL for *S. epidermidis*, and 1250–1450 µg/mL for *B. subtilis*), was added to nutrient broth in microtiter plates. The plates were incubated at 37 °C, and optical density was recorded at 600 nm every 6 h. The specific concentration ranges selected for each strain were based on the MIC estimates previously determined in Section 2.4. Chloramphenicol at a concentration of 60 µg/mL was employed as the positive control. Three independent trials were conducted to determine the subinhibitory concentrations.

### Bactericidal Effect of Postbiotic

The bactericidal activity of the postbiotic was assessed against six bacterial strains by introducing predetermined amounts of the postbiotic into actively growing cultures of the target organisms, according to [21] with slight modifications. For each assay, 5 mL of sterile nutrient broth was inoculated with 500 µL of the bacterial suspension (10⁸ CFU/mL). Freshly prepared postbiotic solutions were sterilized using a 0.2 μm filter and applied at different concentrations: 950–1200 µg/mL for *K. pneumoniae*, 1850–2100 µg/mL for *E. coli*, 1550–1800 µg/mL for *P. aeruginosa*, 1250–1500 µg/mL for *S. aureus*, 950–1200 µg/mL for *S. epidermidis*, and 1550–1800 µg/mL for *B. subtilis*. The inoculated tubes were incubated at 37 °C for 24 h, after which samples were plated onto nutrient agar (NB, BD Difco) to determine bacterial survival, expressed as log N (CFU/mL).

### Microbiological Analysis of Semen Samples

The microbiological analysis of the examined semen was performed following the method of [39] with slight modifications. Briefly, 1 mL of raw semen was diluted in tubes containing 9.0 mL of PBS (0.1 M phosphate buffer with 0.15 M NaCl, pH 7.3) to prepare serial dilutions (10–10^3^). From each dilution, 1 mL was centrifuged at 1500 rpm for 15 min at room temperature. After removing the supernatant, the pellet was resuspended in 100 µL of sterile buffered peptone water (CM0509, Oxoid). This process enhances bacterial detection by concentrating the bacteria in the pellet and removing seminal plasma, which may inhibit bacterial growth. The pellet was then spread onto various culture media: Nutrient Agar for total viable aerobic counts, spore-forming bacteria, and psychrophilic bacteria, and MacConkey Agar for total *coliform* bacteria. All plates were incubated at 37 °C for 48 h, except for the psychrophilic bacteria, which were incubated at 7 °C for 5 days. For counting aerobic spore-forming bacteria, samples were heated in a thermostatically controlled water bath at 80 °C for 10 min, cooled to 37 °C, then diluted and plated on nutrient agar. These were incubated at 37 °C for 48 h. The result expressed by log (N/N0) CFU/mL represents the logarithmic ratio of the current colony-forming units (CFU) in a sample (N) to the initial CFU (N0), used to measure microbial decay.

### Gas Chromatography-Mass Spectrometry (GC-MS) Analysis

Using gas chromatography-mass spectrometry (GC-MS) according the method described by [40], postbiotic *E. coli* Nissle 1917 was analyzed. The analysis was performed using a Thermo Scientific GC-MS system, which included a Gas Chromatography System (1310) and an MS (TSQ 9000). The system was equipped with an HP-5 MS fused silica column. For the GC-MS analysis, helium was used as the carrier gas at a constant flow rate of 1.0 mL/min. The temperature settings were as follows: Ion source (250 °C), Interface (300 °C), and at 300 °C, a 1 µL sample was injected in split mode with a split ratio of 1:50. The column’s temperature program began at 60 °C and was held for 5 min before increasing to 150 °C at a rate of 5 °C/minute. After holding the temperature at 150 °C and increasing it at a rate of 5 °C/minute up to 250 °C (as you mentioned previously), the temperature was further increased to 250 °C at a rate of 30 °C/minute and held for 5 min. The total elution time was 52.67 min. The relative percentage of each component was determined by comparing its average peak area to the total peak area. MS solution software was used to control the system and acquire data.

### Assessment of DPPH Radical Scavenging Activity and Total Phenolic and Flavonoid Contents in Postbiotic

The TPC (total phenolic content) of the postbiotic extract was assessed using the Folin–Ciocalteu (F–C) assay, following the procedures outlined by [41]. The TFC (total flavonoid content) was quantified through the aluminum chloride colorimetric method, as previously described by [42]. The antioxidant capacity of the samples was assessed using the DPPH radical scavenging assay, with ascorbic acid as the reference standard [43].

### Animals and Semen Collection

Ethical approval for all procedures including sampling, animal handling and welfare, was acquired from the Animal Care and Use Committee of Mansoura University (MU-ACUC, AGR.RP.24.10.2). The study adhered to Directive 2010/63/EU and was conducted in accordance with the ARRIVE guidelines 2.0, ensuring the protection of animals used for scientific purposes. A total of 40 semen ejaculates were collected from five water buffalo bulls (*Bubalus bubalis*) aged 4–5 years, housed at the breeding station in Mahalet Mussa near Sakha, Kafr El-Sheikh Governorate, Egypt. The bulls were kept under standard management and feeding conditions. Semen samples were collected weekly over an eight-week period using the artificial vagina method at a temperature of 40–42 °C. The collected samples were promptly transported to the laboratory for analysis. The semen samples were evaluated for progressive motility, live sperm percentage, sperm abnormality rate, and sperm cell concentration. The criteria for acceptable values were set at ≥ 75% for progressive motility, ≥ 70% for live sperm, ≤ 15% for sperm abnormality, and ≥ 800 million/mL for sperm cell concentration. These parameters were used as guidelines for subsequent freezing experiments.

### Semen Extender Preparation, and Experimental Design

Following the method provided by [44] with some modifications, the extender was prepared using standard construction measures at the Center of Artificial Insemination and Embryo Transfer. To prepare the freezing extender, 3.028 g of Tris, 1.675 g of citric acid, 1.25 g of fructose, and 20 mL of egg yolk were dissolved in double-distilled water. Then, 6% glycerol, 100 IU/mL of penicillin and 100 µg/mL of streptomycin were added before bringing the final volume up to 100 mL. Before adding cryoprotectants, we used an osmometer to check the extender’s osmolality, which ranged from 280 to 300 mOsmol, and a pH meter to confirm the pH value was between 6.8 and 6.9. The extender was gently swirled and pre-warmed to 37 °C in a water bath to ensure it was ready for immediate semen dilution. This extender was used as a standard or control extender. Semen samples were diluted and extended with various levels of prepared postbiotic of *E. coli* Nissle 1917 at five doses, including 0 (control extender), 250 µg/mL, 500 µg/mL, 1000 µg/mL, and 2000 µg/mL.

After dilution, the semen was gently shaken and placed in a 37 °C water bath before being gradually cooled to 5 °C. This cooling process (the equilibration period) lasted for four hours before the semen was loaded into 0.25 mL straws. The straws were cooled in liquid nitrogen vapor for 10 min and then submerged in liquid nitrogen. The semen was cryopreserved for 4 weeks before being thawed at 37 °C for 30 s in a water bath for evaluation. The samples were assessed in equilibrated buffalo bull semen, after thawing in a water bath and incubation at 37 °C with 5% CO_2_ for 2 h.

### Semen Parameters Evaluation

#### Progressive Motility

Using a phase-contrast microscope equipped with a heated stage set to 37 °C, we evaluated the percentage of progressive sperm motility for each semen sample. Small aliquots of the diluted sperm (10 µL) were placed on pre-warmed glass slides and covered with a glass coverslip [45]. Progressive motility was assessed by examining 4–5 different fields of view under the microscope for each semen sample. The final motility value was recorded as the average of these successive observations [46].

#### Viability and Abnormalities

According to the protocol outlined by [47], sperm viability was evaluated using the one-step eosin-nigrosin staining technique. This method involved mixing 10 µL of each sample with 5% eosin and 10% nigrosin stain on a glass slide. A minimum of 200 sperm cells from each sample were examined for viability under a light microscope at 400x magnification. Sperm cells with a white or unstained head were categorized as viable (living), while those with a red-stained head were considered dead. Furthermore, the number of sperm cells displaying head and tail morphological abnormalities was recorded using the same magnification as previously described by [48].

#### Plasma Membrane Integrity

To evaluate the membrane integrity of spermatozoa, hypo-osmotic swelling (HOS) assessment was conducted as described by [44]. The HOS solution, with an osmolality of 75 mOsmol/L, consists of a solution containing 6.75 g/L fructose and 3.67 g/L sodium citrate. In brief, 50 µL of semen was mixed with 500 µL of the HOS solution and incubated at 37 °C for 30 min in a water bath. After incubation, ten microliters of the mixture were placed on a microscope slide and covered with a coverslip. A total of 200 spermatozoa were examined for their swelling capacity in the HOS solution. Spermatozoa with swollen or coiled tails were considered to have an intact plasma membrane [49].

#### Computer Assisted Sperm Analysis (CASA)

The CASA technique provides accurate assessment of various sperm motility parameters, which is essential because motility is a reliable indicator of sperm function. Post-thawed semen samples (approximately 0.25 mL each) were evaluated using a CASA system (Sperm Vision, Minitube, Tiefenbach, Germany). The system integrated an Olympus BX microscope connected to a rapid-scan digital camera. All assessments were performed at a controlled temperature of 37 °C, capturing images at a rate of 60 frames per second with 4x dark-field optics. Approximately 1500 spermatozoa were evaluated per treatment to ensure a sufficient sample size for statistical analysis. The motion characterization was recorded, including distance average path (DAP, µm), distance curved line (DCL, µm), velocity average path (VAP, µm/sec), distance straight line (DSL, µm), velocity curved line (VCL, µm/sec), linearity (LIN = VSL/VCL), velocity straight line (VSL, µm/sec), straightness (STR = VSL/VAP), wobble (WOB = VAP/VCL), amplitude of lateral head displacement (ALH, µm), and beat cross frequency (BCF, Hz).

#### Valuation of Acrosome Reaction

Two hundred microliters of post-thawed semen from each sample were placed into a plastic tube, containing an equal volume of 0.2% trypan blue. The tube was then incubated for 10 min at 37 °C in a water bath. After incubation, the spermatozoa were diluted with 2 mL of modified Brackett and Oliphant medium without albumin [50], and centrifuged at 700 g for 6 min. The supernatant was discarded, and the sperm in the pellet was resuspended in 2–3 mL of the same media and centrifuged as before. This step was repeated until the suspension was clear or pale blue. A 10–20 µl aliquot of the sperm suspension was placed on a slide and smeared with a second glass slide. The smears dried rapidly on a warming stage (40 °C).

The sperm on the slides were then stained for 35–40 min using a 10% solution of Giemsa stock in distilled water. This staining solution was prepared immediately before use. Next, the stained slides were rinsed under a stream of distilled water and air-dried. To determine the variability and acrosome reaction, under brightfield microscopy, about 100 spermatozoa were arbitrarily chosen per slide and observed as follows: (a) Live sperm with intact acrosome: Post-acrosome is white, and the acrosome is bright pink/purple. (b) Live sperm with detached acrosome: Post-acrosome is white, and the acrosome is white (true acrosome reaction). (c) Dead sperm with intact acrosome: Post-acrosome is blue or dark blue, and the acrosome is dark pink/purple. (d) Dead sperm with detached acrosome: Post-acrosome is blue, and the acrosome is white/gray-white (False acrosome reaction).

#### Flow Cytometry

We used Annexin V staining to assess the status of apoptotic sperm, following the method described by [51]. Post-thawed semen samples (*n* = 3 for each group, approximately 1 mL each) were suspended in a 2 mL binding buffer and thoroughly mixed. Subsequently, 100 µL of the suspension was combined with 5 µL of PI (phycoerythrin label) and 5 µL of Annexin V (fluorescein isothiocyanate label). The mixture was then incubated in a dark environment at room temperature for a minimum of 15 min.

Flow cytometry was used to analyze the incubated sperm cells. The cells were suspended in 200 µL of binding buffer before being analyzed with an Accuri C6 Cytometer and its accompanying software. Finalizing the analysis, we determined the percentages of cells that were negative or positive for Annexin V (A-/A+) and propidium iodide (PI-/PI+).

Following the classification by [52], examined sperm were categorized into four groups:

- Viable cells (A-/PI-): These presented no fluorescence signal, indicating no membrane dysfunction.
- Early apoptotic cells (A+/PI-): These were viable cells labeled with Annexin V but not PI.
- Late apoptotic cells (A+/PI+): These were dead cells stained with both Annexin V and PI, indicating a damaged, permeable membrane.
- Necrotic cells (A-/PI+): These were dead cells stained only with PI, signifying a complete loss of membrane integrity.

The number of cells in each category was determined based on their forward and side scatter characteristics.

#### Antioxidant Related Makers in Extender of Post-Thawed Buffalo Bull Semen

The levels of MDA (malondialdehyde), TAC (total antioxidant capacity), and NO (nitric oxide) were measured in the extender by centrifuging the post-thawed semen samples for 10 min at 6000 rpm. Kits from Bio-diagnostic (Giza, Egypt) were used to assess these antioxidant-related biomarkers using a colorimetric method following the manufacturer’s instructions. The kit numbers were TA 25 13, MD 25 29, and NO 25 33 for TAC, MDA, and NO, respectively. All assays were conducted using a spectrophotometer (Spectro UV-Vis Auto, UV-2602; Labomed, Los Angeles, CA, USA).

#### Sperm Ultrastructure Changes Using Transmission Electron Microscopy

Using transmission electron microscopy (TEM, JEM-2100 F electron microscope, JEOL Ltd., Tokyo, Japan), the ultrastructural changes in buffalo bull sperm cells were examined following the detailed protocol described by [44]. The post-thawed semen samples were first fixed in 2% glutaraldehyde in PBS for 2–3 h. Subsequently, they were washed three times with PBS via centrifugation (5 min at 4 °C). The samples were then post-fixed in 1% osmium tetroxide for approximately two hours at 4 °C before being dehydrated with acetone and embedded in Epon resin. Thin sections of the prepared samples were cut using an RMC ultramicrotome. The sections were then stained with uranyl acetate and lead citrate before being examined with a TEM. To capture the images, the TEM was equipped with Digital Micrograph Gatan and Soft Imaging Spectator software. We selected the fields for imaging randomly.

#### Fertility Rate

A total of 100 multiparous buffalo cows, showing spontaneous estrus, were randomly selected and divided into two groups. The first group served as the control group, while the second group received a postbiotic treatment at a concentration of 2000 µg/mL, which was determined to be the most effective treatment based on in vitro outcome parameters. Each group consisted of 50 females. All cows were artificially inseminated with frozen/thawed semen by a single AI technician. The 56-day non-return rate (56-NRR) was recorded.

### Statistical Analysis

First, we conducted Levene’s test and the Shapiro-Wilk test to confirm that the numerical data met the assumptions of homogeneity of variance and normality. Subsequently, we analyzed the data using a one-way analysis of variance (ANOVA) in SAS to assess the impact of the various treatments. Upon observing a significant difference in the ANOVA results, we employed Tukey’s test to pinpoint specific variations between the treatment groups, with a significance level established at *p* < 0.05.

## Results

### Antibacterial Activity of Postbiotic

**Fig. 2:**
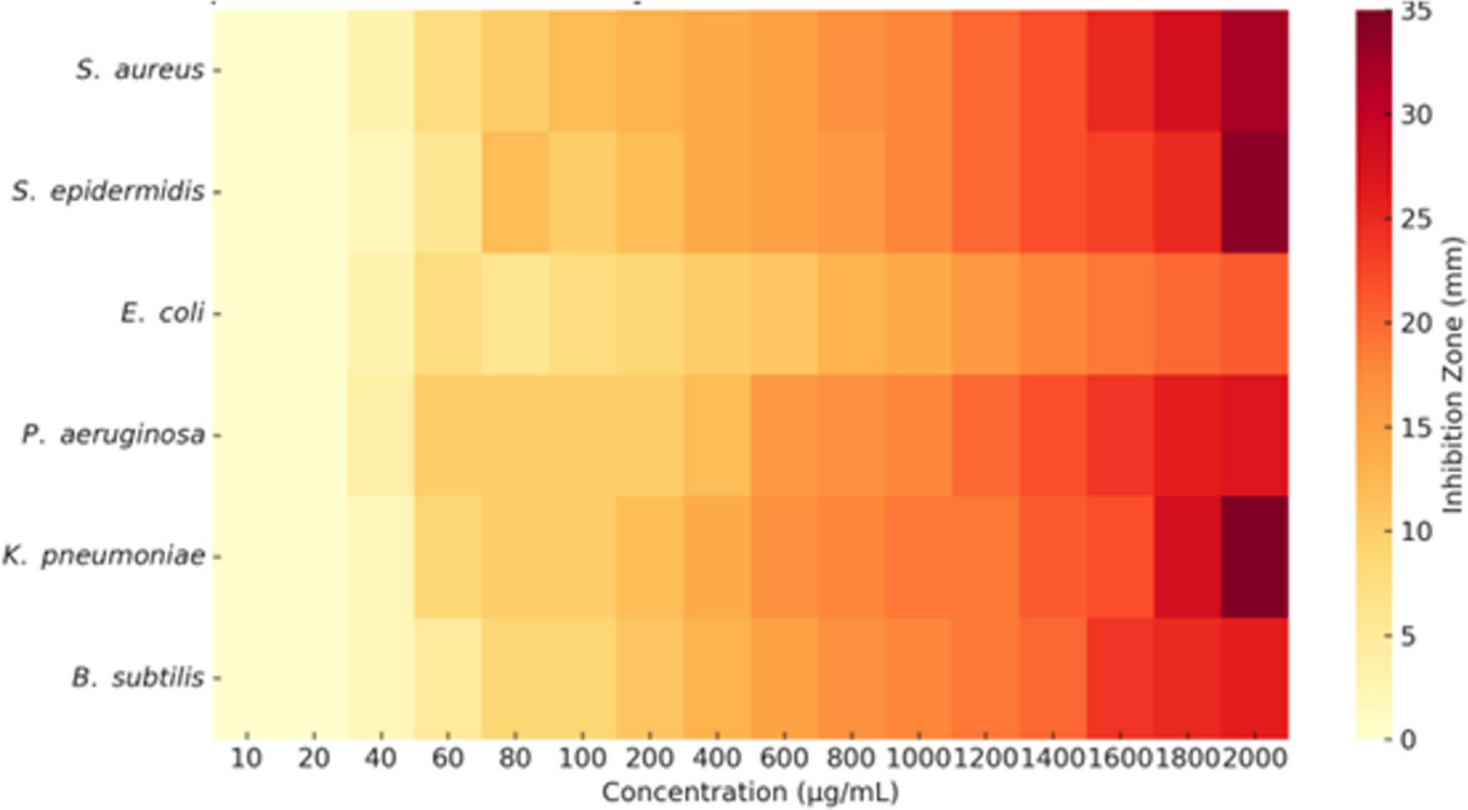
Heatmap of antibacterial activity across concentrations. Darker colors indicate stronger activity at higher concentrations. light yellow = 0–5 mm, orange = 10–15 mm, dark red = ≥ 30 mm

The heatmap illustrates the antibacterial activity of the postbiotic against six bacterial strains (*E. coli*,* P. aeruginosa*,* K. pneumoniae*,* S. aureus*,* S. epidermidis* and *B. subtilis)* across a concentration gradient (10–2000 µg/mL) (Fig. 2). No inhibition was observed at concentrations ≤ 20 µg/mL, while measurable inhibition zones appeared from 40 µg/mL onward, confirming a dose-dependent response. Among the tested organisms, *K. pneumoniae* exhibited the highest susceptibility, reaching an inhibition zone of 35 mm at 2000 µg/mL, followed by *S. epidermidis* (34 mm) and *S. aureus* (32 mm). The spore-forming *B. subtilis* demonstrated moderate inhibition (26 mm at 2000 µg/mL), while *P. aeruginosa* showed similar but slightly lower sensitivity (27 mm). In contrast, *E. coli* displayed the weakest inhibition response, with a maximum zone of only 21 mm at the highest concentration, highlighting its relative tolerance compared to the other strains.

### Bacteriostatic Effect of Postbiotic

**Fig. 3:**
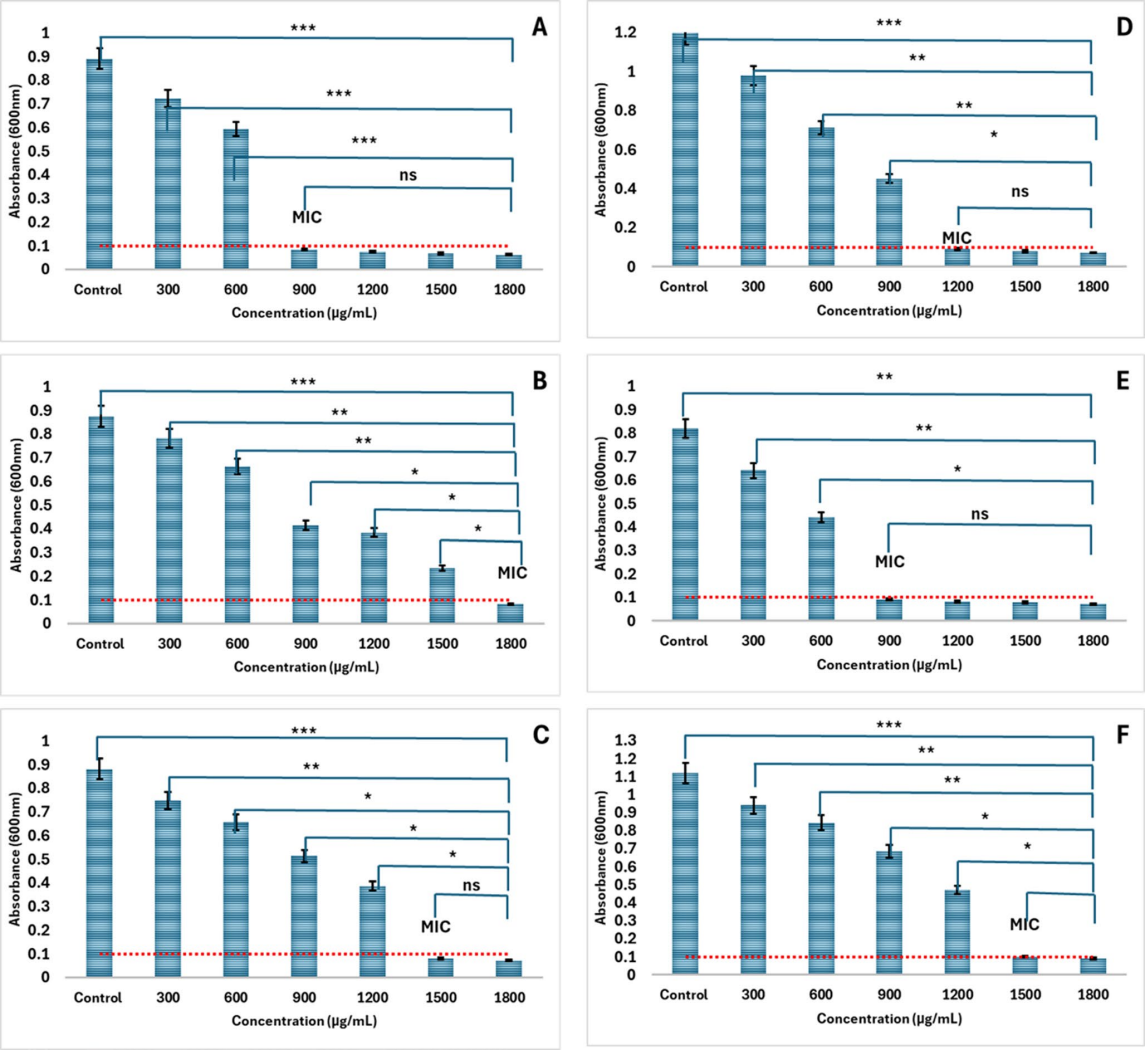
The minimum inhibitory concentration (MIC) of the postbiotic was determined against six indicator strains such as *K. pneumoniae* (**A**), *E. coli* (**B**), *P. aeruginosa* (**C**), *S. aureus* (**D**), *S. epidermidis* (**E**), and *B. subtilis* (**F**). The untreated group served as positive control. Arrows indicate the MIC values for each organism. Statistical significance was assessed using one-way ANOVA with Tukey’s multiple comparisons, with thresholds defined as ***p < 0.001, *p < 0.01, *p < 0.05*, and ns indicating no significance

The MIC of the postbiotic was determined against six indicator strains such as *K. pneumoniae* (900 µg/mL, Fig. 3A), *E. coli* (1800 µg/mL, Fig. 3B), *P. aeruginosa* (1500 µg/mL, Fig. 3C), *S. aureus* (1200 µg/mL, Fig. 3D), *S. epidermidis* (900 µg/mL, Fig. 3E), and *B. subtilis* (1500 µg/mL, Fig. 3F) using absorbance at 600 nm as a measure of bacterial growth (Fig. 3). A concentration-dependent reduction in absorbance was observed across all strains, with lower MIC values reflecting higher inhibitory capacity (*p* < 0.001). Accordingly, *K. pneumoniae* and *S. epidermidis* demonstrated the greatest sensitivity (*p* < 0.001), while *E. coli* exhibited the highest tolerance (*p* < 0.001), requiring the largest concentration to reach inhibition. The absorbance of blank broth was 0.050 ± 0.005; thus, absorbance values greater than 0.1 were considered positive for bacterial growth in microtiter plate assays.

### Subinhibitory Dose of Postbiotic

**Fig. 4:**
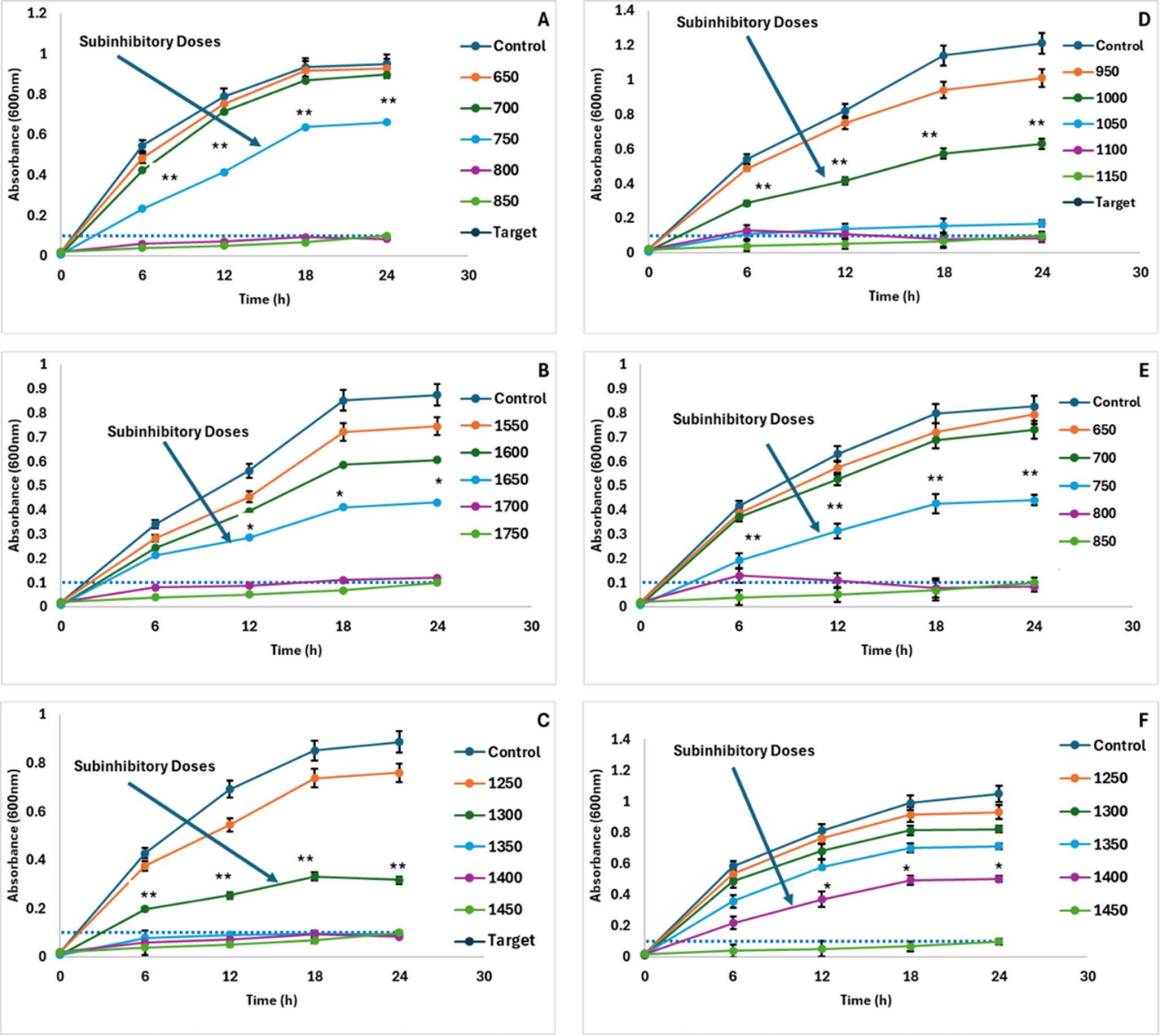
The growth curve and subinhibitory doses of *K. pneumoniae* (**A**), *E. coli* (**B**), *P. aeruginosa* (**C**), *S. aureus* (**D**), *S. epidermidis* (**E**), and B. *subtilis* (**F**) strains were determined in relation to postbiotic concentrations in nutrient broth. Statistical significance was assessed using one-way ANOVA with Tukey’s multiple comparisons, where **p < 0.01, *p < 0.05, and ns indicates no significance

The sub-inhibitory concentrations (sub-MICs) of the postbiotic were evaluated against six indicator strains (Fig. 4) such as *K. pneumoniae* (750 µg/mL, Fig. 4A), *E. coli* (1700 µg/mL, Fig. 4B), *P. aeruginosa* (1300 µg/mL, Fig. 4C), *S. aureus* (1000 µg/mL, Fig. 4D), *S. epidermidis* (750 µg/mL, Fig. 4E), and *B. subtilis* (1400 µg/mL, Fig. 4F) based on growth kinetics. The growth curves demonstrated a concentration-dependent suppression of bacterial proliferation, with progressive delays in growth onset and reduced biomass accumulation at increasing sub-MIC levels. Notably, *K. pneumoniae* (*p* < 0.01), and *S. epidermidis* (*p* < 0.01) exhibited strong growth suppression at relatively low sub-MICs (750 µg/mL), confirming their high sensitivity to the compound. *S. aureus* also showed marked inhibition at 1000 µg/mL, consistent with its relatively low MIC value (*p* < 0.05). In contrast, *E. coli* required the highest sub-MIC (1700 µg/mL) to achieve measurable suppression, highlighting its higher tolerance compared to other strains. *P. aeruginosa* (*p* < 0.05) and *B. subtilis* (*p* < 0.05) demonstrated intermediate responses at 1300 µg/mL and 1400 µg/mL, respectively, showing delayed growth but not complete inhibition at early time points. Statistical analysis indicated significant differences in bacterial growth compared to controls, with inhibitory effects most pronounced within the first 12 h. Collectively, these results emphasize that the postbiotic exerts time- and dose-dependent growth-suppressive effects, with distinct variability across Gram-negative, Gram-positive, and spore-forming bacteria.

### Bactericidal Effect of Postbiotic

**Fig. 5:**
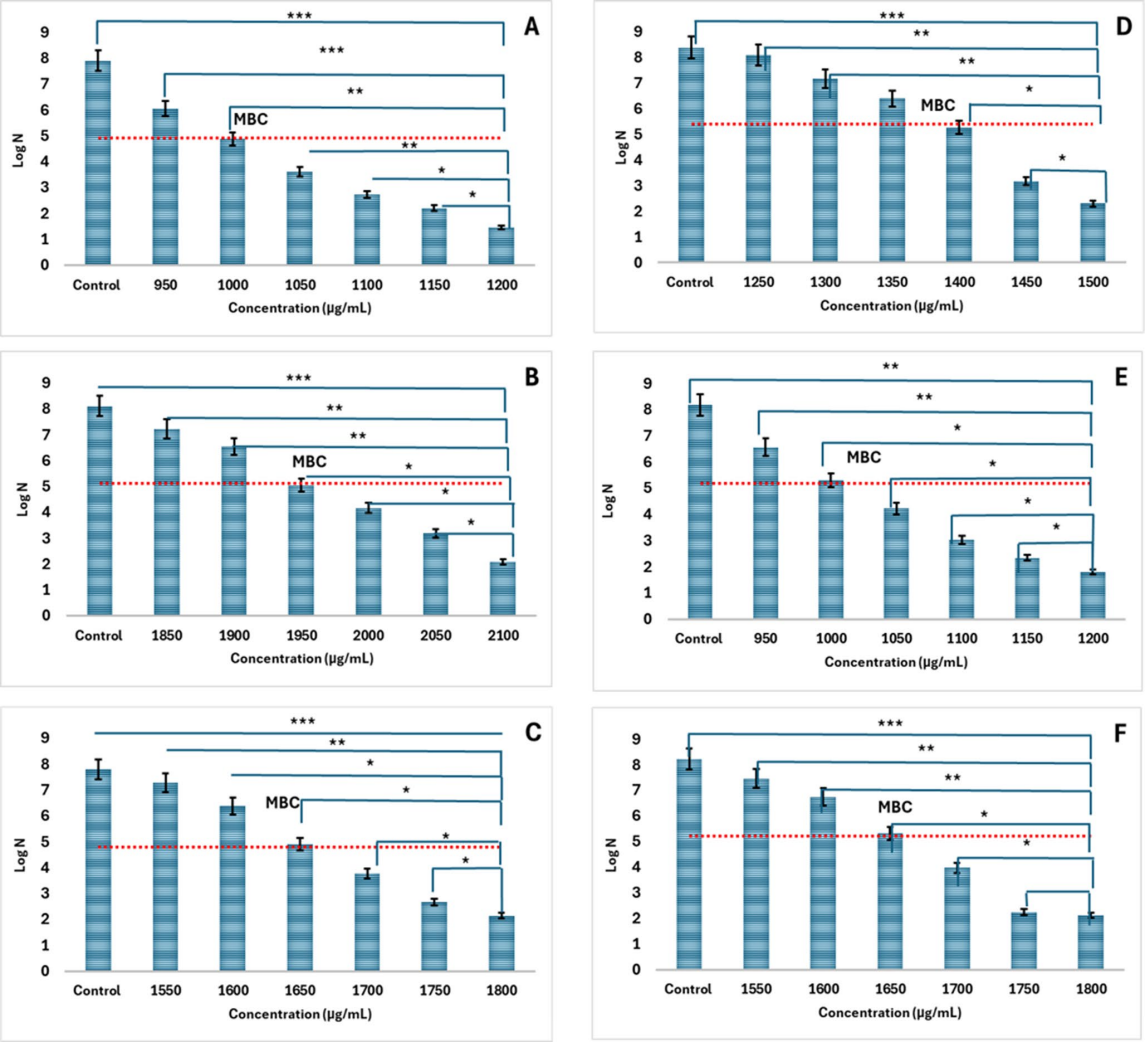
Minimum bactericidal concentration (MBC) of the postbiotic was determined against six indicator strains such as *K. pneumoniae* (**A**), *E. coli* (**B**), *P. aeruginosa* (**C**), *S. aureus* (**D**), *S. epidermidis* (**E**) and *B. subtilis* (**F**) using the CFU assay. The untreated group served as positive control. Arrows indicate the MBC values for each organism, while the dotted line in panels (**A**–**F**) represents the 3-log₁₀ reduction threshold. ***p < 0.001, **p < 0.01, *p < 0.05, and ns indicates no significance

The bactericidal effect obtained by the postbiotic showed a clear dose-dependent trend for the six strains tested at 37 °C (Fig. 5). The differing MBCs, defined as the lowest concentration able to achieve ≥ 3 log^10^ reduction in viable counts, characterized the varying susceptibilities of the strains. *K. pneumoniae* (*p* < 0.001) and *S. epidermidis* were the most sensitive, with an MBC of 1000 µg/mL, followed by *S. aureus* at 1400 µg/mL. For *P. aeruginosa* and *B. subtilis*, 1650 µg/mL was necessary to reach the same reduction in viability, while *E. coli* was the most resistant to killing, with an MBC of 1950 µg/mL.

### Concentration-Dependent Effects of Postbiotic on Semen Microbiological Quality

**Fig. 6:**
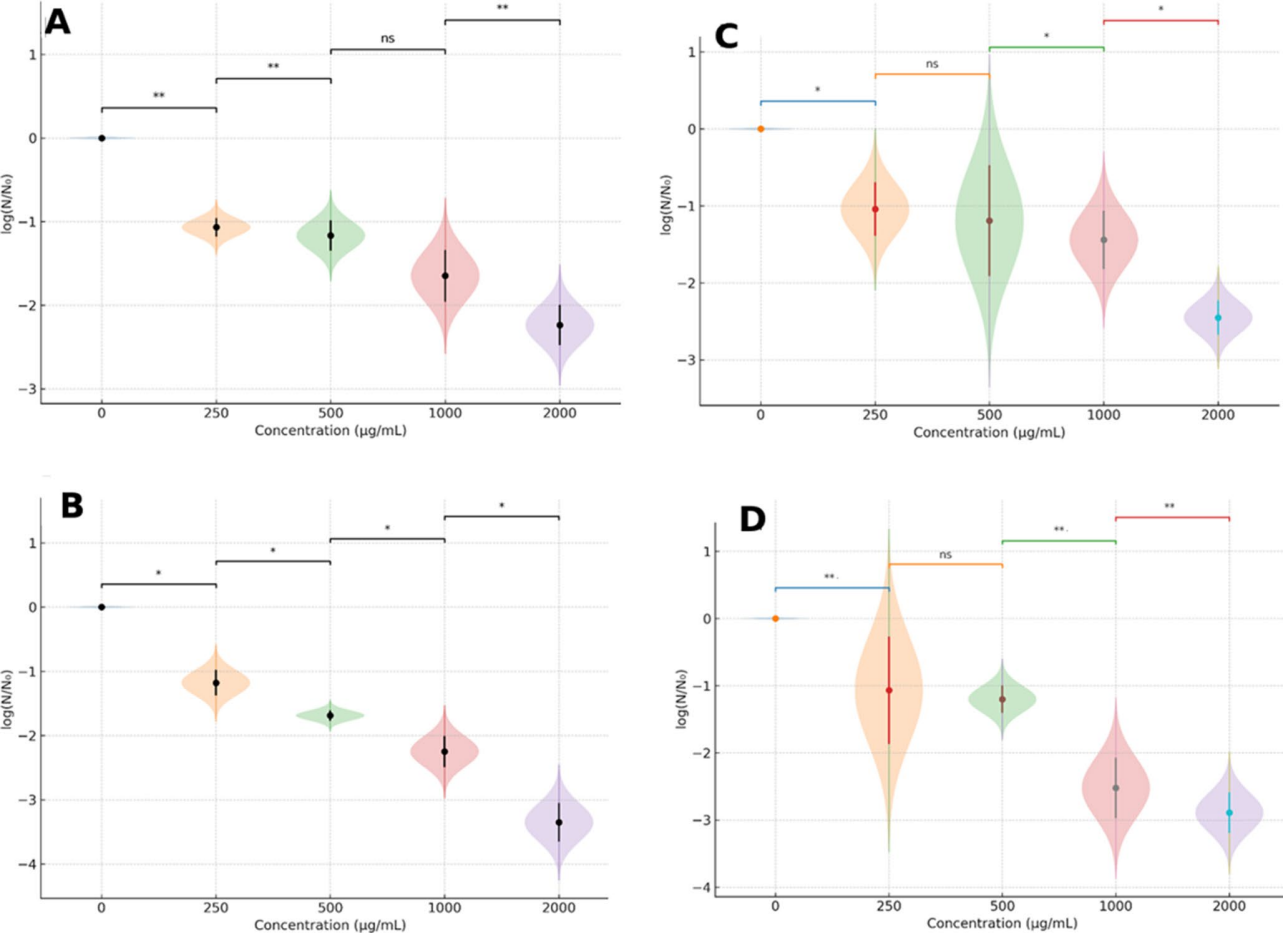
Effect of postbiotic on the microbiological attributes of post-thawed semen such as total bacterial count (**A**), spore-forming bacteria (**B**), coliforms (**C**), and psychrophilic bacteria (**D**). Statistical significance was assessed using one-way ANOVA with Tukey’s multiple comparisons, where **p < 0.01, *p < 0.05, and ns indicates no significance

The obtained results demonstrated that the application of postbiotic at different concentrations had a marked effect on improving the microbiological quality of semen, as the results were expressed as log^₁₀^(N/N₀). As shown in Fig. 6A, the total bacterial count decreased significantly in a concentration-dependent manner (*p* < 0.01), with clear significant differences between most concentrations. However, there was no statistically significant difference between adding 500–1000 µg of postbiotics per mL (*p* > 0.05). Spore-forming bacteria (Fig. 6B) were significantly reduced in all groups of semen enriched with postbiotic compared to the control groups (*p* < 0.01). For *coliforms* (Fig. 6C), the results indicate lower levels of *coliforms* in extended semen fortified with postbiotic, except for the dose of 500 µg. The levels of psychrophilic bacteria (Fig. 6D) in post-thawed semen were significantly reduced in all probiotic groups compared to the control group (*p* < 0.01). However, the addition of 500 µg did not produce significant differences (*p* > 0.05). These findings suggest that postbiotic supplementation can effectively reduce various groups of microorganisms in semen, especially at higher concentrations. This can improve the hygienic quality and preservation potential of semen.

### Analysis of Postbiotic by Gas Chromatography-Mass Spectrometry (GC/MS)

**Fig. 7:**
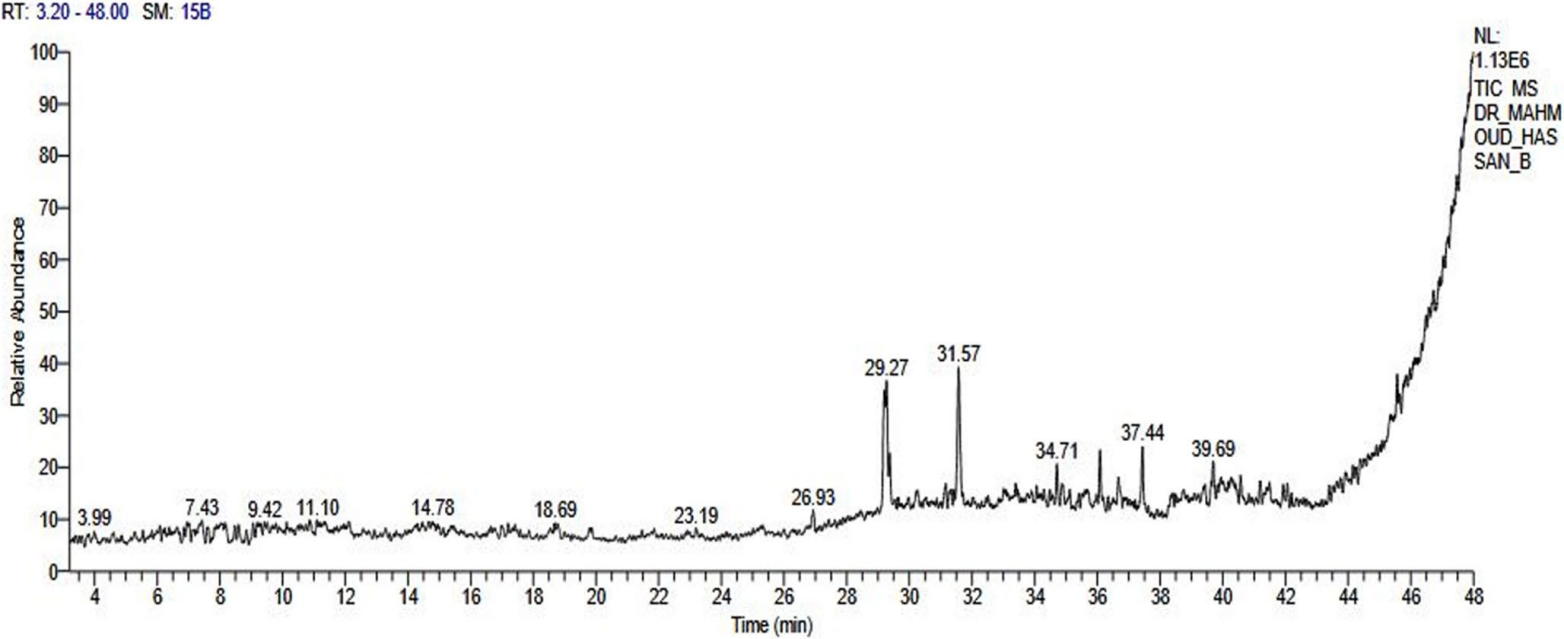
Analysis of gas chromatography-mass spectrometry (GC/MS) of postbiotic components

According to the GC/mass analysis of postbiotic (Table 1 and Fig. 7), mainly phytochemical compounds were identified, including docosanoic acid, 1,2,3-propanetriyl ester (0.90%), stearic acid, 3-(octadecyloxy)propyl ester (1.24%), 1-methyl-2-pyrrolidineethanol (1.49%), 4(3 H)-pyrimidinone/1 H-imidazole-4,5-dihydro-2-methyl (0.96%/0.87%), 10-octadecenoic acid methyl ester and similar C18 fatty acid methyl esters (3.74%), cyclohexanol, 1R-4-acetamido-2,3-cis-epoxy (3.34%), 2-myristynoyl-glycinamide (1.85%), 1-monolinoleoylglycerol trimethylsilyl ether (1.81%), and glycine, N-3,5,7,12-tetrakis(trimethylsiloxy)cholan-24-yl derivative (0.82%).

**Table 1:** The principal compounds were detected in the postbiotic based on GC/mass analysis

No	RT (min)	Compound Name	Molecular Formula	Area %	Comments
1	7.59	Docosanoic acid, 1,2,3-propanetriyl ester	C_69_H_134_O_6_	0.90	Fatty acid ester, typical bacterial metabolite
2	33.40	Stearic acid, 3-(octadecyloxy)propyl ester	C_39_H7_8_O_3_	1.24	Lipid-derived ester. Possibly bacterial in origin
3	35.12	1-Methyl-2-pyrrolidineethanol	C_7_H_15_NO	1.49	Could arise from amino-acid degradation (proline-derived).
4	35.67/35.71	4(3 H)-Pyrimidinone/1 H-Imidazole-4,5-dihydro-2-methyl	C4H_4_N_2_O/C_4_H_8_N_2_	0.96/0.87	Nitrogenous bases/nucleotide degradation products. Possible bacterial origin.
5	36.08	10-Octadecenoic acid, methyl ester (and similar C18 FAMEs)	C_19_H_36_O_2_	3.74	Fatty acid ester, typical bacterial metabolite
6	36.66	Cyclohexanol, 1R-4-acetamido-2,3-cis-epoxy	C_8_H_13_NO_3_	3.34	Possible microbial transformation product
7	39.40	2-Myristynoyl-glycinamide	C_16_H_28_N_2_O_2_	1.85	Amino acid derivative, microbial metabolite
8	39.95	1-Monolinoleoylglycerol trimethylsilyl ether	C_27_H_54_O_4_Si_2_	1.81	Glycerol ester derivative, bacterial metabolite
9	41.42	Glycine, N-3,5,7,12-tetrakis(trimethylsiloxy)cholan-24-yl derivative	C_36_H_69_NO_6_Si_3_	0.82	Bile acid derivative or microbial conjugate

### Antioxidant Activity and Bioactive Compound Content of *E. Coli* Nissle 1917 Postbiotics

**Fig. 8:**
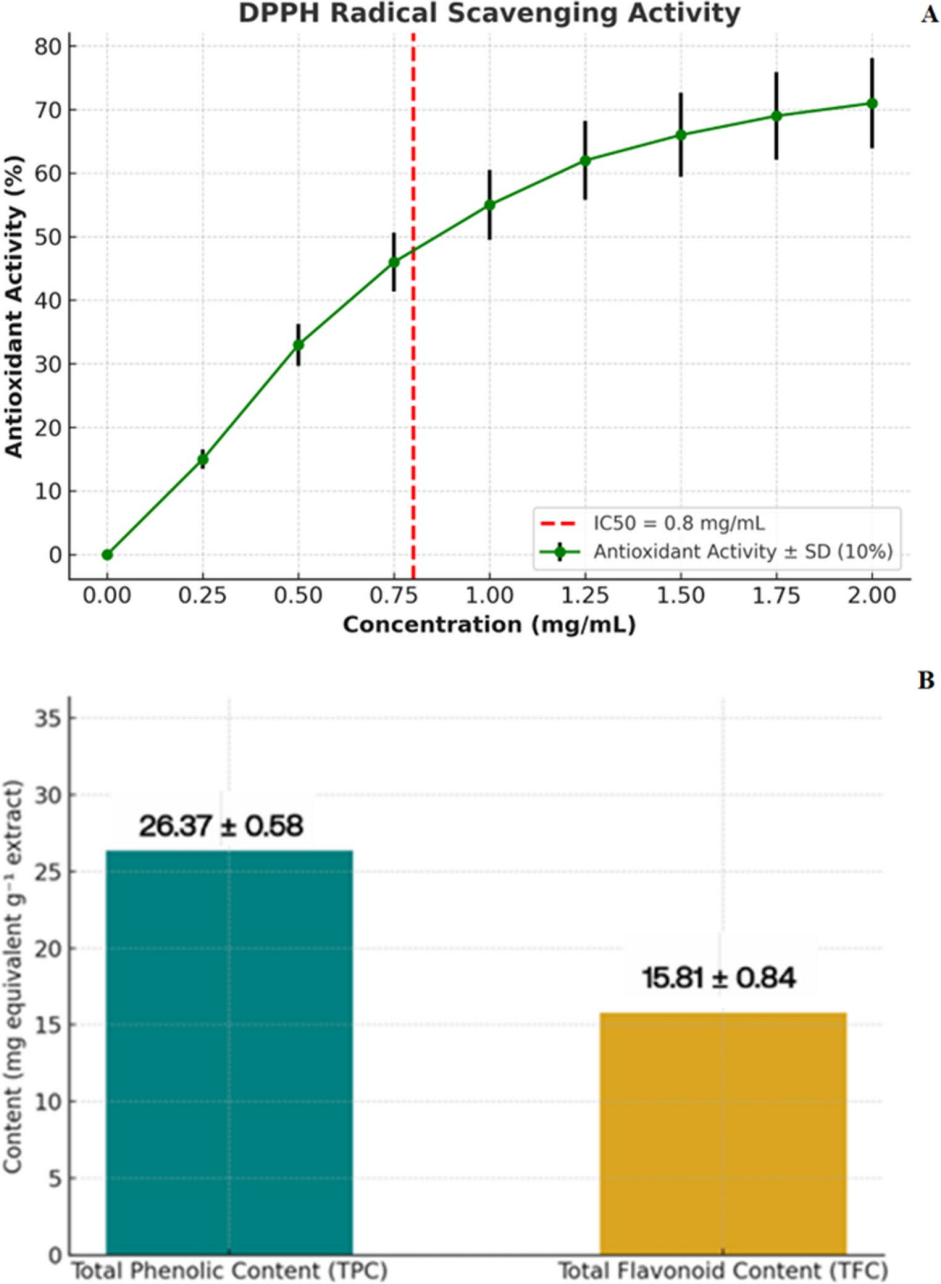
DPPH radical scavenging activity (**A**) and bioactive compound content (**B**) of postbiotic produced from *E. coli* Nissle 1917

The DPPH radical scavenging activity of the postbiotic produced from *E. coli* Nissle 1917 showed a clear concentration-dependent increase, with higher concentrations leading to greater free radical inhibition (Fig. 8). The half-maximal inhibitory concentration (IC₅₀) was estimated at 0.80 mg/mL, which is the concentration needed to achieve 50% scavenging of DPPH radicals (Fig. 8A). The total phenolic content (TPC) and total flavonoid content (TFC) of the same postbiotic were quantified (Fig. 8B). The TPC was found to be 26.37 mg gallic acid equivalent per gram of extract, while the TFC was 15.81 mg catechin equivalent per gram of extract. These values indicate a significant presence of phenolic and flavonoid compounds, which are known for their antioxidant and redox properties. Overall, these results suggest that the antioxidant activity of *E. coli* Nissle 1917 postbiotics may be closely linked to their phenolic and flavonoid composition, highlighting their potential as natural antioxidant agents.

### Effects on Semen Attributes of Buffalo Bull after Equilibration (5 °C for 4 h)

The impact of different levels of postbiotic on sperm quality is detailed in Table 2. The addition of 1000–2000 µg/mL of postbiotic significantly enhanced progressive motility (*p* < 0.01), sperm viability (*p* < 0.01), and membrane integrity (*p* < 0.01) compared to the control group. These enhancements resulted in a 13.0% and 15.12% increase in sperm motility, an 11.6% and 12.0% increase in sperm viability, and a 10.3% and 14.5% increase in membrane integrity, respectively. There was no significant impact on sperm abnormality with the addition of postbiotics at all tested levels (*p* > 0.05). Moreover, the inclusion of 250–500 µg/mL showed slight improvement, but these changes were not statistically significant compared to other groups (*p* > 0.05).

**Table 2:** Sperm variable percentage as affected by supplementing Tris-extender with postbiotic in buffalo bull semen after equilibration (5 °C for 4 h)

Extender	Sperm characteristics (%)			
	Progressive motility	Viability	Membrane integrity	Abnormality
Control	76.7 ± 1.67^b^	79.7 ± 1.76^b^	77.7 ± 1.45^b^	7.7 ± 0.88
Postbiotic 250 µg/mL	81.7 ± 1.67^ab^	83.3 ± 1.45^ab^	83.7 ± 1.67^ab^	7.0 ± 1.15
Postbiotic 500 µg/mL	83.3 ± 1.67^ab^	85.3 ± 0.88^ab^	83.7 ± 0.67^ab^	6.3 ± 0.88
Postbiotic 1000 µg/mL	86.7 ± 1.67^a^	89.0 ± 1.00^a^	88.0 ± 2.52^a^	6.3 ± 0.88
Postbiotic 2000 µg/mL	88.3 ± 1.67^a^	89.3 ± 1.45^a^	89.0 ± 1.53^a^	7.7 ± 0.33
*P value*	0.005	0.002	0.005	0.680

^a−b^ Means denoted with different superscripts in each column are significantly different at *p* < 0.05

### Effects on Post-Thawed Semen Attributes of Buffalo Bull

In the case of post-thawed parameters, it was shown that adding 2000 µg/mL of postbiotic had the greatest percentages of enhanced progressive motility (*p* < 0.001), sperm viability (*p* < 0.001), and membrane integrity (*p* < 0.001) compared to the control group (Table 3). All postbiotic tested levels significantly improved all investigated parameters (except for sperm abnormality compared to the control group (*p* < 0.01). However, adding 250 µg/mL postbiotic exhibited the lowest values in all postbiotic groups, while the 500–1000 µg/mL exhibited similar results for all investigated parameters indicating intermediate values in postbiotic groups.

**Table 3:** Sperm variable percentage as affected by supplementing Tris-extender with postbiotic in post-thawed buffalo bull semen

Extender	Sperm characteristics (%)			
	Progressive motility	Viability	Membrane integrity	Abnormality
Control	33.8 ± 0.82^c^	35.0 ± 1.20^c^	34.6 ± 0.75^c^	10.0 ± 0.57
Postbiotic 250 µg/mL	38.8 ± 0.82^b^	41.0 ± 1.22^b^	40.0 ± 0.82^b^	9.1 ± 0.48
Postbiotic 500 µg/mL	41.3 ± 0.82^ab^	42.6 ± 0.98^ab^	41.1 ± 1.04^ab^	8.6 ± 0.46
Postbiotic 1000 µg/mL	42.5 ± 0.94^ab^	44.4 ± 1.21^ab^	42.3 ± 0.80^ab^	9.9 ± 0.72
Postbiotic 2000 µg/mL	43.8 ± 1.25^a^	46.9 ± 1.06^a^	44.0 ± 0.98^a^	9.5 ± 0.53
***P value***	< 0.0001	< 0.0001	< 0.0001	0.410

^a,−c^ Means denoted with different superscripts in each column are significantly different at *p* < 0.05

### Effects on Sperm Quality of Kinematic Parameters of Post-Thawed Buffalo Bull Sperm

The addition of 1000–2000 µg/mL of postbiotic significantly improved the values of PM (*p* < 0.001), DSL (*p* < 0.001), VSL (*p* < 0.001), STR (*p* < 0.001), LIN (*p* < 0.001), and BCF (*p* < 0.001) compared to the control, 250, and 500 µg/mL postbiotic groups (Table 4).The values of DAP (*p* < 0.0001), DCL (*p* < 0.05), VAP (*p* < 0.05), and VCL (*p* < 0.05) were the highest in the 2000 µg/mL postbiotic group. There were no statistical variances amongst the control group and the addition of 250 and 500 µg/mL postbiotic for VCL, DSL, VAP, VSL, LIN, WOB, and BCF (*p* > 0.05). The value of WOB (%) was the greatest in 1000 µg/mL postbiotic group (*p* < 0.05).

**Table 4:** Effect of supplementing Tris-extender with postbiotic on kinematic parameters of post-thawed buffalo bull sperm

Item			Postbiotic (µg/mL)			*P *Value
	Control	250	500	1000	2000	
PM (%)	34.2 ± 1.13^c^	39.1 ± 1.14^b^	42.9 ± 0.88^b^	44.0 ± 0.82^a^	47.0 ± 1.10^a^	< 0.0001
DAP (µm)	18.4 ± 0.35^c^	19.7 ± 0.71^bc^	20.5 ± 0.77^ab^	21.7 ± 0.65^ab^	22.7 ± 0.66^a^	0.001
DCL (µm)	27.9 ± 0.45^b^	31.0 ± 1.13^ab^	30.6 ± 1.52^ab^	30.6 ± 1.13^ab^	33.0 ± 1.04^a^	0.048
DSL (µm)	13.3 ± 0.41^b^	14.0 ± 0.41^b^	14.9 ± 0.66^b^	17.8 ± 0.63^a^	18.5 ± 0.53^a^	< 0.0001
VAP (µm/sec)	40.9 ± 0.79^b^	45.7 ± 1.58^ab^	46.1 ± 1.81^ab^	46.6 ± 1.24^ab^	49.8 ± 1.55^a^	0.004
VCL (µm/sec)	61.7 ± 1.11^b^	71.8 ± 2.65^ab^	68.9 ± 3.59^ab^	65.7 ± 2.40^ab^	72.3 ± 2.47^a^	0.04
VSL (µm/sec)	29.5 ± 0.88^b^	32.5 ± 0.90^b^	33.5 ± 1.50^b^	38.2 ± 1.14^a^	40.5 ± 1.25^a^	< 0.0001
STR (%)	71.6 ± 1.27^b^	70.6 ± 0.69^b^	72.0 ± 0.87^b^	81.9 ± 1.03^a^	81.0 ± 0.79^a^	< 0.0001
LIN (%)	47.1 ± 1.06^b^	45.0 ± 0.95^b^	48.3 ± 1.32^b^	57.9 ± 1.30^a^	55.7 ± 0.81^a^	< 0.0001
WOB (%)	65.7 ± 0.42^c^	63.1 ± 0.74^bc^	66.7 ± 1.41^bc^	70.7 ± 0.99^a^	68.4 ± 0.48^ab^	< 0.0001
ALH (µm)	1.6 ± 0.08^c^	2.2 ± 0.09^b^	2.3 ± 0.07^b^	2.5 ± 0.06^b^	3.1 ± 0.05^a^	< 0.0001
BCF (Hz)	24.5 ± 0.84^b^	24.0 ± 0.71^b^	24.9 ± 0.71^b^	28.4 ± 1.17^a^	30.1 ± 0.51^a^	< 0.0001

DCL, distance curved line (µm); DAP, distance average path (µm); DSL, distance straight line (µm); VCL, velocity curved line (µm/sec); VAP, velocity average path (µm/sec); VSL, velocity straight line (µm/sec); LIN, linearity (VSL/VCL); STR, straightness (VSL/VAP); WOB, wobble (VAP/VCL); BCF, beat cross frequency (Hz) and ALH, amplitude of lateral head displacement (µm)

^a−c^ Means denoted with different superscripts in each row are significantly different at *p* < 0.05

### Effects on Acrosome Status of Post-Thawed Buffalo Bull Sperm

The addition of all postbiotic tested resulted in higher live sperm with intact acrosome compared to the control group (Table 5). In contrast, all postbiotic levels pointedly decreased the percentages of live sperm with detached acrosome (*p* < 0.05), dead sperm with intact acrosome, and dead sperm with detached acrosome compared to the control treatment (*p* < 0.01). This designates that postbiotic sustained the acrosome structure of bull sperm.

**Table 5:** Effect of supplementing Tris-extender with postbiotic on acrosome reaction of post-thawed buffalo bull semen

Treatment	Live sperm with intact acrosome	Live sperm with detached acrosome	Dead sperm with intact acrosome	Dead sperm with detached acrosome
Control	34.4 ± 0.93^b^	28.6 ± 1.03^a^	32.0 ± 1.52^a^	5.0 ± 0.45^a^
Postbiotic 250 µg/mL	48.0 ± 0.84^a^	23.4 ± 1.50^b^	25.4 ± 1.21^b^	3.2 ± 0.37^b^
Postbiotic 500 µg/mL	49.4 ± 1.60^a^	23.6 ± 0.68^b^	23.2 ± 1.46^b^	2.8 ± 0.37^b^
Postbiotic 1000 µg/mL	51.6 ± 0.98^a^	21.8 ± 1.07^b^	23.6 ± 1.08^b^	3.0 ± 0.45^b^
Postbiotic 2000 µg/mL	52.0 ± 0.71^a^	21.0 ± 0.63^b^	24.4 ± 0.81^b^	2.6 ± 0.40^b^
***P value***	0.001	0.0004	0.004	0.003

^a-b^ Means denoted within the same column with different superscripts are significantly different at *p* < 0.05

### Effects on Post Thawed Buffalo Bull Semen Incubated at 37 °C and 5% CO_2_ for 2 h

Adding postbiotics to buffalo freezing media at levels of 500, 1000, and 2000 µg/mL significantly improved sperm progressive motility (*p* < 0.001) and membrane integrity (*p* < 0.001) compared to the group without postbiotics (Table 6). A lower concentration of 250 µg/mL showed no significant effect on these parameters. While the 250 µg/mL dose had similar results to other groups for most parameters (except for viability), a concentration of 2000 µg/mL yielded the best viability values. Interestingly, there was no significant change in viability between the 2000, 1000, and 500 µg/mL groups (*p* > 0.05). The addition of postbiotics had no effect on the abnormality rate of the sperm (*p* > 0.05).

**Table 6:** Sperm variable percentage as affected by supplementing Tris-extender with postbiotic in post thawed buffalo bull semen incubated at 37 °C and 5% CO_2_ for 2 h

Extender	Sperm characteristics (%)			
	Progressive motility	Viability	Membrane integrity	Abnormality
Control	31.9 ± 0.91^b^	33.9 ± 0.81^c^	30.4 ± 0.62^b^	11.8 ± 0.59
Postbiotic 250 µg/mL	35.0 ± 1.34^ab^	36.8 ± 1.06^bc^	34.9 ± 1.29^ab^	11.1 ± 0.55
Postbiotic 500 µg/mL	38.8 ± 1.25^a^	40.3 ± 1.52^ab^	37.3 ± 1.51^a^	11.4 ± 0.46
Postbiotic 1000 µg/mL	39.4 ± 1.48^a^	41.3 ± 1.33^ab^	38.5 ± 1.38^a^	10.6 ± 1.10
Postbiotic 2000 µg/mL	40.0 ± 1.64^a^	42.4 ± 1.66^a^	39.8 ± 1.37^a^	11.0 ± 1.00
***P value***	0.0004	0.0003	< 0.0001	0.88

^a−c^ Means denoted with different superscripts in each column are significantly different at *p* < 0.05

### Effects on Antioxidant Biomarkers in Post-Thawed Buffalo Bull Semen

The addition of all postbiotics tested resulted in higher total antioxidant capacity (TAC) levels (mM/L) compared to the free postbiotic group (*p* < 0.0001) (Table 7). These increases were 35.6%, 37.01%, 52.05%, and 63.01%, respectively, compared to the control group. The doses of 1000 and 2000 µg/mL of postbiotics significantly reduced the levels of malondialdehyde (MDA) compared to the control group (*p* < 0.01). There was no statistical difference between the addition of 250–500 µg/mL for MDA and 250 µg/mL for nitric oxide (NO) compared to other groups (*p* > 0.05). For NO, the addition of 500 µg/mL and higher levels resulted in lower values than those of the control group (*p* < 0.001). Adding postbiotics improved TAC and reduced oxidative stress markers in post-thawed bull semen.

**Table 7:** Effect of supplementing Tris-extender with postbiotic on total antioxidant capacity, oxidative and nitrosative biomarkers in post-thawed buffalo bull semen

Treatment	TAC (mM/L)	MDA (nmol/mL)	NO (µmol/L)
Control	0.73 ± 0.04^b^	14.8 ± 0.73^a^	8.5 ± 0.63^a^
Postbiotic 250 µg/mL	0.99 ± 0.06^a^	13.9 ± 0.46^ab^	6.8 ± 0.29^ab^
Postbiotic 500 µg/mL	1.00 ± 0.03^a^	13.5 ± 0.37^ab^	6.1 ± 0.37^b^
Postbiotic 1000 µg/mL	1.11 ± 0.08^a^	11.9 ± 0.34^b^	5.4 ± 0.41^b^
Postbiotic 2000 µg/mL	1.19 ± 0.05^a^	11.4 ± 1.07^b^	5.2 ± 0.45^b^
*P value*	0.0001	0.008	0.0002

^a−b^ Means denoted within the same column with different superscripts are significantly different at *p* < 0.05. TAC (Total antioxidant concentration), MDA(Malondialdehyde) and NO (Nitric oxide)

### Effects on Apoptosis Like Changes

All supplemented postbiotic groups significantly increased the percentages of viable sperm, with the highest values observed in the 2000 µg/mL postbiotic groups (*p* < 0.001) (Table 8). Conversely, supplementing freezing media with postbiotics led to a decrease in the percentages of apoptotic buffalo sperm (*p* < 0.001). The lowest levels of necrotic sperm were observed in frozen semen supplemented with 2000 µg/mL postbiotics compared to other groups (*p* < 0.001).

**Table 8:** Effect of supplementing Tris-extender with postbiotic on apoptosis-like changes of post-thawed buffalo bull sperm (Annexin V/PI assay)

Treatment	Apoptosis like changes (%)		
	Viable(A−/PI−)	Apoptotic (A+/PI+)	Necrotic (A−/PI+)
Control	19.8 ± 1.55^d^	69.4 ± 2.02^a^	10.8 ± 0.50^b^
Postbiotic 250 µg/mL	30.9 ± 4.13^c^	51.3 ± 4.02^b^	17.9 ± 0.12^a^
Postbiotic 500 µg/mL	49.87 ± 3.07^b^	37.1 ± 2.41^c^	13.0 ± 0.67^b^
Postbiotic 1000 µg/mL	52.0 ± 3.18^b^	35.4 ± 2.51^c^	12.5 ± 0.67^b^
Postbiotic 2000 µg/mL	64.6 ± 2.75^a^	33.8 ± 2.78^c^	1.6 ± 0.03^c^
***P value***	< 0.0001	< 0.0001	< 0.0001

^a-d^ means denoted within the same column with different superscripts are significantly different at *p* < 0.05

### Effects on Sperm Ultrastructure

**Fig. 9:**
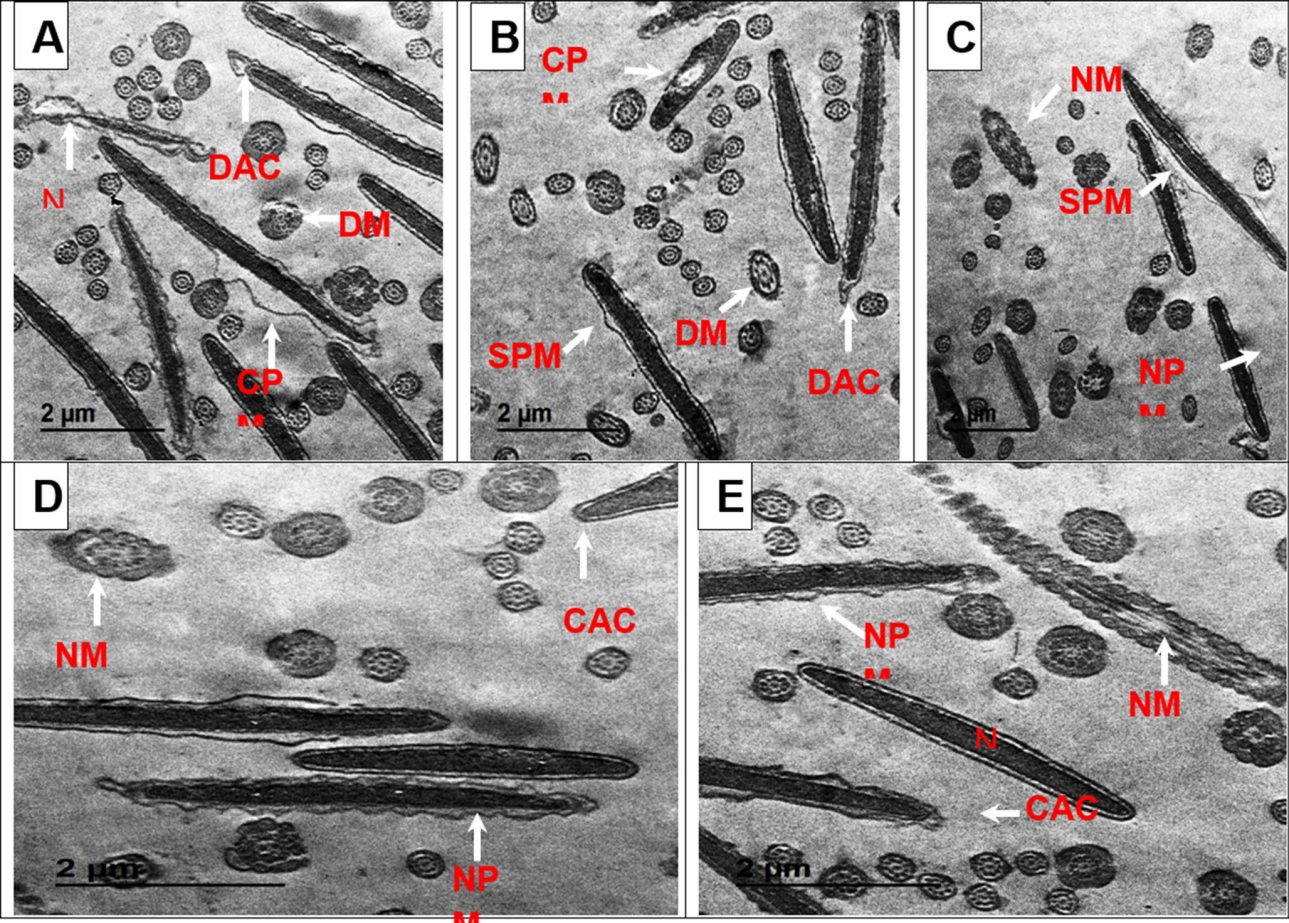
the Impact of postbiotic on post-thawed buffalo sperm ultra-structural with various concentrations of postbiotic (0, 250, 500, 1000 and 2000 µg/mL). In the semen samples of the control group (Fig. **A**), the nucleus (N), slightly damaged acrosome (DAC), and observed damaged mitochondria (DM) indicated complete damage to the acrosome of bullfrog sperm. However, the addition of 250 µg/mL (Fig. **B**) or 500 µg/mL (Fig. **C**) Postbiotic showed a slight improvement in sperm ultrastructure, as evidenced by a slightly dilated plasma membrane (SPM) and normal mitochondria. Furthermore, adding 1000 µg/mL (Fig. **D**) or 2000 µg/mL (Fig. **E**) Postbiotic exhibited more improvements in sperm ultrastructure, with normal mitochondria (NM), intact acrosome (NAC), and normal plasma membrane (NPM)

In the control group semen samples (Fig. 9A), the buffalo sperm exhibited complete damage to the acrosome, evidenced by the appearance of the nucleus (N), a slightly damaged acrosome (DAC), and damaged mitochondria (DM). The postbiotic led to dose-dependent enhancements in sperm ultrastructure. The lower doses (250–500 µg/mL; Fig. 9B and C) showed only a slight improvement, evidenced by a slightly dilated plasma membrane (SPM) and normal mitochondria. However, the higher doses (1000–2000 µg/mL; Fig. 9D and E) resulted in more pronounced improvements, featuring an intact acrosome (NAC), normal mitochondria (NM), and a normal plasma membrane (NPM).

### Pregnancy Rate

**Fig. 10:**
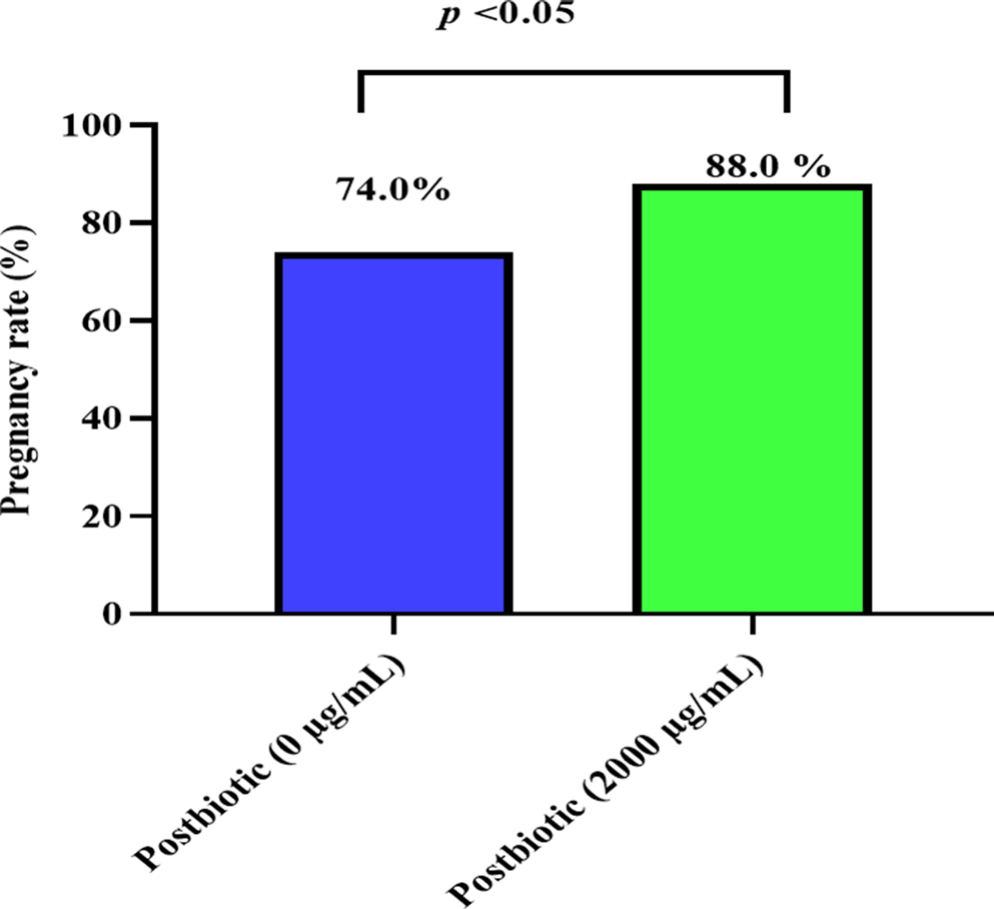
The pregnancy rates in buffalo cows inseminated with semen were compared between the control group (without postbiotic) and the group enriched with postbiotic (2000 µg/mL)

Based on the results of in vitro studies, the authors recommended a dose of 2000 µg of postbiotic/mL for insemination. The pregnancy rate was 74% in the control group and 88% in the treated group (Fig. 10). The inclusion of 2000 µg/mL postbiotic significantly enhanced the pregnancy rates compared to the group without the additive (control group, *p* < 0.05).

## Discussion

Postbiotics are gaining favor over probiotics because they offer superior therapeutic benefits such as improved metabolic, immune, and antioxidant functions along with significant advantages. They are safer and more cost-effective to produce and store, and they remain stable in a wider range of environments, making them easier to use.

Postbiotics, including those derived from *E. coli* Nissle 1917, exhibit notable antimicrobial activity due to the production of bioactive metabolites such as bacteriocins, organic acids, and peptides. *E. coli* Nissle 1917 naturally synthesizes microcins H47 and M, which play a significant role in its ability to inhibit the growth of various pathogenic bacteria by disrupting bacterial membranes and interfering with cellular processes. Recent studies also demonstrate that postbiotics from different probiotic strains can produce inhibition zones against a range of pathogens, and their antimicrobial effects are considered a promising alternative to conventional antibiotics in semen extender systems, reducing antibiotic usage and associated resistance risks.

The effectiveness of postbiotics as antimicrobial agents depends on specific strain characteristics and production conditions, supporting their application in preservation strategies for reproductive biotechnology [53–56]. The scope of this research was the isolation and biological evaluation of the postbiotic synthesized by *E. coli* Nissle 1917. Specifically, we investigated its antimicrobial potential including both bactericidal and bacteriostatic action, its antioxidant activity, and the identification of key bioactive molecules. Consistent with our results, the novel probiotic strain, *Lactobacillus plantarum* TW29-1, has been shown to exhibit dual functionality, providing both therapeutic health benefits and valuable preservation properties for the food industry [57]. The antimicrobial properties of the postbiotic derived from *E. coli* Nissle 1917 were evaluated using a panel of bacterial pathogens. Based on the observed antimicrobial activity, follow-up experiments were performed to determine the postbiotic’s potential for use as a therapeutic agent against infectious bacteria.

Studies have shown that yogurt fortified with the *E. coli* Nissle 1917 probiotic exhibits broad-spectrum antimicrobial activity against numerous bacterial pathogens, including *E. cloacae*, *K. pneumoniae*, *E. coli*, *P. vulgaris*, and *P. aeruginosa* [21]. Specifically, inhibition zones for these bacteria ranged from 21.3 to 26.7 mm at a concentration of 21.3 to 26.7 mm at 100 µg/mL. The same authors also reported the anti-fungal activity of the *E. coli* Nissle 1917 postbiotic. Furthermore, the postbiotic has been successfully utilized as a therapeutic treatment for intestinal mucositis due to its anti-inflammatory action, which upregulates the mRNA expression of the mucin MUC2 and the tight junction protein TJP1 [58].

The compounds identified as metabolites of *E. coli* Nissle 1917 demonstrate varying potential for antioxidant and antimicrobial activities, supporting the strain’s probiotic effects. Fatty acid esters such as docosanoic acid derivatives and stearic acid esters are known to disrupt microbial membranes, exhibiting broad antimicrobial properties while also contributing to oxidative stability in biological membranes [57]. Nitrogenous bases and amino acid derivatives including pyrimidinone, imidazole, and myristynoyl-glycinamide possess radical scavenging and antimicrobial activities, which can protect host tissues from oxidative damage and inhibit pathogen proliferation. Additionally, bile acid derivatives modulate gut microbial communities and have both membrane-disrupting antibacterial effects and antioxidant functions that attenuate inflammation. Cyclohexanol and glycerol esters contribute further to microbial antagonism and bioactive metabolite diversity. These collective activities corroborate the beneficial role of *E. coli* Nissle 1917 in maintaining gut health through direct antimicrobial action and modulation of oxidative stress, aligning well with experimental findings on its immunomodulatory and protective properties [21, 59, 60].

Semen cryopreservation often leads to a significant increase in pathogenic microbes. Introducing an antimicrobial agent can mitigate this risk by reducing microbial load and improving sperm viability [61]. The inclusion of probiotic *E. coli* Nissle 1917 significantly reduced the total bacterial count, total *coliform *count, spore-forming bacteria, and psychrophilic bacteria in post-thawed semen samples. These results are in line with the previous results provided in the above section. This effect may be attributed to the peptides within the postbiotic compounds. These peptides directly compromise the cell membranes of pathogenic bacteria, resulting in leakage and subsequent cell death [62]. It has been shown in current research that curcumin nanoparticles reduce the semen microbiota in buffaloes [39].

To further explore the beneficial effects of postbiotic *E. coli* Nissle 1917, we added the postbiotic to cryo-media of buffalo sperm. The study indicated that adding 1000–2000 µg/mL significantly enhanced sperm quality, including progressive motility, sperm viability, membrane integrity, and kinematic parameters such as PM, DSL, VSL, STR, LIN, and BCF. It also improved intact acrosome, viable sperm, preserved sperm ultrastructure, and enhanced the pregnancy rate. This improvement was accompanied by a decrease in oxidative stress markers (MDA and NO) and the percentages of apoptotic buffalo sperm. This study is the first to elucidate the beneficial effects of a postbiotic derived from *E. coli* Nissle 1917 on sperm function in buffalo. These findings are consistent with some previous studies. For example, dietary supplementation with a postbiotic from *Lactiplantibacillus plantarum SNI3* (LbSNI3) significantly improved ejaculate volume and sperm concentration in roosters [19]. Another study showed that a postbiotic derived from lactic acid bacteria reduced the rate of abnormalities and improved acrosome integrity in rabbit sperm, although it did not significantly affect sperm motility or viability [22]. Conversely, high doses of *L. rhamnosus PB01* (DSM 14870) postbiotic had a significant negative impact on the quality of human sperm in an in vitro study [8]. Pyrroloquinoline Quinone (PQQ), a compound found in *E. coli* Nissle 1917, is believed to enhance sperm motility by increasing the supply of ATP. It achieves this by serving as a cofactor for several redox enzymes, which support mitochondrial function and, consequently, enhance ATP synthesis [63]. This link between PQQ and increased ATP production underscores its potential as a supplement for enhancing male fertility. In line with our results, secretions from *L. plantarum* exhibited a cryoprotective effect on sperm motility when included in the sperm freezing medium [64]. In addition to microbial control, the use of *E. coli* Nissle 1917 postbiotics enhanced key sperm parameters, including progressive motility, viability, and membrane integrity. This echoes the cryoprotective effects described in studies utilizing *L. plantarum* secretions for human sperm [64]. While motility preservation was the primary benefit observed with *L. plantarum*, the broader scope of improvement in buffalo sperm, including acrosome integrity and various kinematic traits, underscores the superior multifunctionality of *E. coli* Nissle 1917 postbiotics. This expanded effect may be attributed to differing postbiotic profiles or the adaptive response of buffalo sperm to these bioactive agents.

The observed increase in total antioxidant capacity and reduction of oxidative stress markers further reinforce the crucial role of postbiotics in mitigating cellular damage during cryopreservation [65]. Similar antioxidant benefits have been reported in studies involving *Bacillus subtilis* and *Saccharomyces cerevisiae* postbiotics in heat-stressed rabbits [66]. It was shown that improved reproductive performance was linked to reduced oxidative stress. However, the current findings provide additional evidence that the antimicrobial action, combined with antioxidant properties, offers a more comprehensive strategy for protecting sperm during the freeze-thaw process. The synergistic relationship between antioxidants and antimicrobial pathways plays a critical role in sperm cryopreservation [67]. Antioxidants neutralize ROS, preventing lipid peroxidation and DNA damage in sperm cells. Simultaneously, antimicrobial agents reduce pathogen-induced oxidative stress and inflammation, thereby limiting further ROS production [35]. Recent studies demonstrate that combining antioxidant and antimicrobial strategies enhances sperm motility, viability, and membrane integrity during freezing and thawing. For example, co-supplementation of glutathione with selenium nanoparticles improved both antioxidant capacity and antimicrobial defense in bull semen extenders, resulting in better cryopreservation outcomes [68]. Additionally, natural compounds with both antioxidant and antimicrobial activities, such as essential oils from *Thymus vulgaris*, have shown promise in improving post-thaw sperm quality. These findings illustrate the integrated protective mechanisms that jointly preserve sperm functionality during cryopreservation [69].

Cryopreservation can reduce the antioxidant defense in sperm cells, so supplementing with natural antioxidants may help support the defensive system and improve the survival of sperm during cooling or freezing cycles. In this study, we discovered that the postbiotic *E. coli* Nissle 1917 demonstrated strong antioxidant capacity with an IC50 value of 0.8 mg/mL. This effect is likely attributed to the extract’s high concentration of beneficial compounds, with 26.37 mg gallic acid equivalent per gram of extract in TPC and 15.81 mg catechin equivalent per gram of extract in TFC. The antioxidant activity observed in this study is consistent with previous research findings [70, 71].

Consistent with our findings, studies have shown that feeding roosters a diet with the *L. plantarum SNI3* (LbSNI3) postbiotic enhanced their testicular levels of TAC and SOD while reducing MDA levels [19]. This effect may be due to a metabolite, γ-glutamyl-glutamate, which can enter the glutathione cycle and bolster the testes’ defense against oxidative stress. Postbiotics derived from *B. subtilis* and/or *S. cerevisiae* significantly reduced levels of MDA and increased antioxidant levels (TAC and GSH-Px) in rabbits [66].

Some researchers suggest that the antioxidant properties of *E. coli* Nissle 1917 postbiotics may be attributed to the presence of PQQ. This potent redox cofactor has demonstrated promising effects in treating and preventing age-related neurodegenerative diseases by scavenging free radicals and reducing oxidative stress. Supplementation with *E. coli* Nissle 1917 led to a reduction in nitric oxide levels in all groups, indicating its ability to suppress peroxynitrite formation and prevent neurotoxicity. Additionally, a study found that including *E. coli* Nissle 1917 in the diets of laying Japanese quail enhanced their antioxidant capacity [20]. *E.coli* Nissle 1917 in laying Japanese quaildiets enhanced antioxidant capacity [23]. Due to its potent antioxidant properties, PQQ isolated from *E. coli* Nissle can neutralize ROS bursts, protecting mitochondrial membranes and metabolic enzymes. This helps counteract age-related mitochondrial dysfunction and preserve metabolic function [63]. Other studies have suggested that the postbiotic *E. coli* Nissle 1917 can enhance mitochondrial activity, as demonstrated in previous research [71]. The enhancement of mitochondrial activity could support ATP supply for sperm motility, as confirmed in this study through sperm kinetic parameters [71].

Given that acrosome integrity is crucial for sperm-ovum penetration [72], this study investigated the effect of the postbiotic on post-thawed buffalo sperm quality. The addition of the postbiotic (1000–2000 µg/mL) significantly improved viability metrics: it increased the percentage of live sperm with intact acrosomes and simultaneously decreased the percentage of dead sperm (both those with intact and detached acrosomes). Overall, a significant increase in viable sperm and a reduction in the proportion of apoptotic sperm were observed across the postbiotic treatment groups. The postbiotic’s capacity to protect acrosome integrity against cryodamage is attributed to its antioxidant and anti-apoptotic action [63].

To gain more insight into the protective mechanism, we assessed the ultrastructural changes in buffalo sperm following postbiotic treatment. Doses of 1000–2000 µg/mL offered a greater protective effect. This cytoprotection was chemically confirmed by a reduction in MDA and NO (markers of oxidative damage) and an increase in the semen’s TAC [5, 8, 43]. Maintaining the integrity of the plasma membrane, acrosome, and mitochondria is vital for optimal sperm function [43], consequently, for enhancing the fertilization rate in animals. Our findings align with previous work showing that *Lactobacillus plantarum* secretions have a cryoprotective effect on human sperm motility, likely due to the protection of the sperm’s cellular structure [64].

Pregnancy rate is considered one of the most important measures of reproductive capacity in buffalo, directly reflecting a cow’s economic value. In this study, we found that adding 2000 µg/mL significantly improved the pregnancy rate in buffalo cows. Multiple studies align with our findings, indicating that postbiotics can enhance reproductive capacity in rabbits [66], and roosters [19]. Postbiotics derived from *Bifidobacteria* have been shown to reduce acute inflammatory responses and gut barrier disruption [73, 74]. This therapeutic effect is achieved by activating specific signaling pathways that bolster innate immune function, which in turn inhibits the colonization of pathogenic bacteria [14, 26]. This mechanism highlights the crucial role of postbiotics in modulating host defense systems to maintain intestinal health. The future role of postbiotics in livestock breeding goes beyond reproductive enhancement; they are becoming essential tools for sustainable and eco-friendly production. Research is focusing on reducing antibiotic use and improving environmental outcomes. Postbiotics function as effective non-antibiotic alternatives by stabilizing gut health and boosting the immune system. Specific components, including short-chain fatty acids (SCFAs), organic acids, peptides, and microbial fragments, are crucial for modulating the gut-gonadal axis in ruminants. This modulation supports more efficient nutrient uptake and metabolism, which is essential for optimizing reproductive health. Postbiotics offer a dual benefit by enhancing animal fertility and performance while reducing the ecological impact of the livestock sector.

## Conclusion

For the first time, this study explores the beneficial antimicrobial and antioxidant effects of postbiotic derived *E. coli* Nissle 1917 on buffalo bull semen. The addition of this postbiotic, at concentrations of 1000–2000 µg/mL, significantly improved sperm quality, movement, and acrosome integrity, and also reduced sperm cell death. These positive results were confirmed by increased TAC and lower levels of MDA and NO. Furthermore, the postbiotic derived *E. coli* Nissle 1917 preserved the sperm’s ultrastructure after cryopreservation, which led to a higher pregnancy rate in buffalo cows inseminated with semen containing 2000 µg/mL of the postbiotic derived *E. coli* Nissle 1917. Postbiotics are becoming increasingly popular due to their scalability and cost-effectiveness. They provide a stable and long shelf-life, which helps reduce logistical barriers and storage costs compared to live supplements. This makes them an economical and reliable method for improving male reproductive function. However, further research is needed to confirm these beneficial effects, especially when integrating the use of omics tools for stronger evidence.

## Data Availability

The data supporting the findings of this study will be made available upon reasonable request to the corresponding authors.
